# Supplementary material for: COVID-19 amplified racial disparities in the US criminal legal system
Source: Nature. 2023 Apr 19;617(7960):344–50. doi: 10.1038/s41586-023-05980-2 (PMC10172107; doi:10.1038/s41586-023-05980-2)
Supplement: Supplementary file 1 — This file contains Supplementary Sections 1–5 and includes Supplementary Figs. 1–23 and Supplementary Tables 1–7. [file 41586_2023_5980_MOESM1_ESM.pdf]

nature portfolio

https://doi.org/10.1038/s41586-023-05980-2

Supplementary information

COVID-19 amplified racial disparities in the US criminal legal system

In the format provided by the authors and unedited

A.1 State-by-state breakdowns

In Figure A.1, we show the prison population over time for all 50 states, the District of Columbia, and the Federal Bureau of Prisons. In Table A.1, we give an overview of the scope of each state's data in our dataset. In Table A.2, we list every state in order of the maximum reduction in prison population, alongside the month that this decrease was observed. In Figure A.2, we reproduce state-specific versions of Figure 1B.

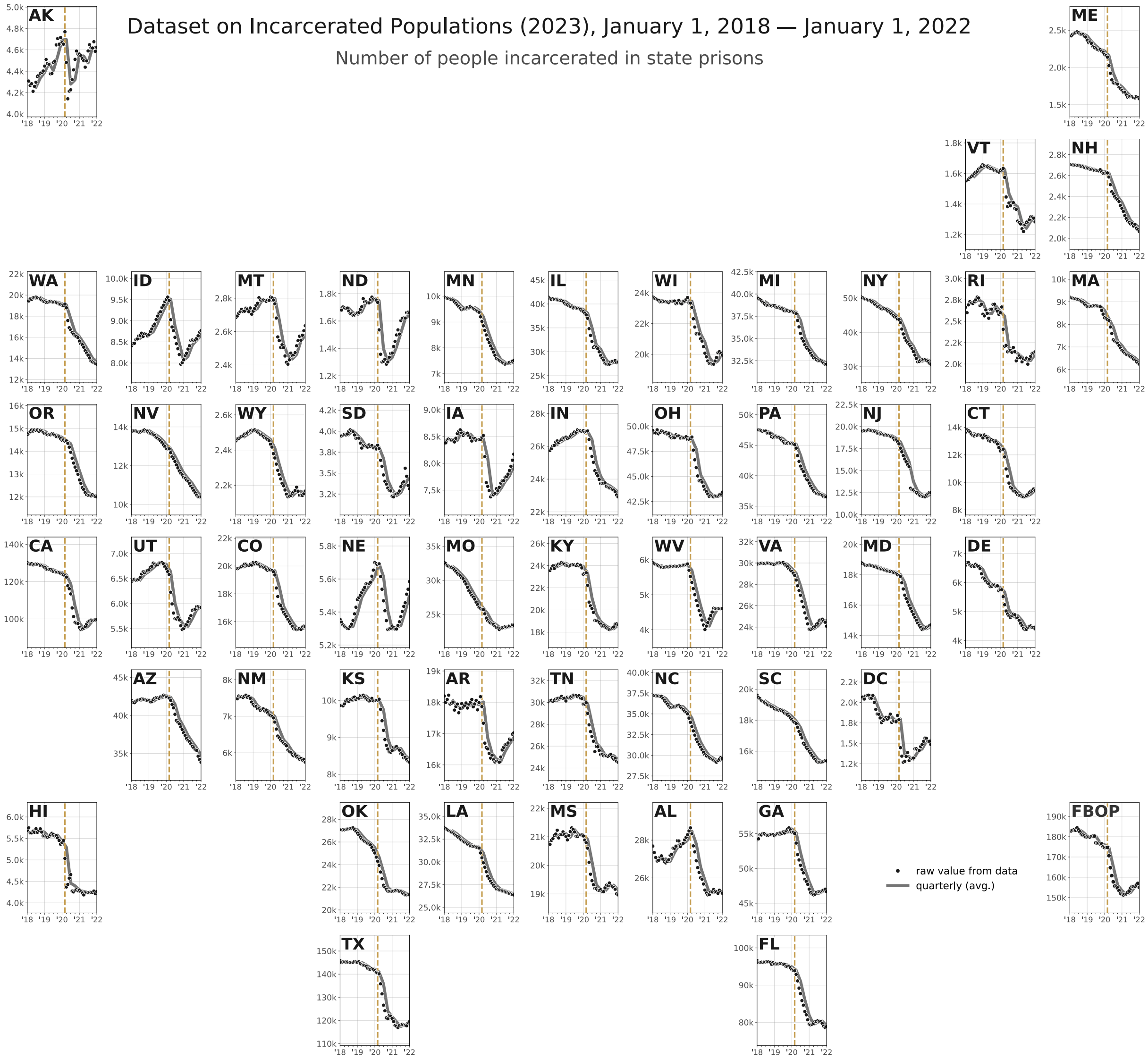

Figure A.1: State-by-state (and federal) time series of prison populations. For consistency, the data plotted have been up- or down-sampled to the quarterly level (most states in the dataset report monthly data, but several report twice-yearly and yearly).

Note that for several states in Figure A.2, we plot the percent incarcerated population who are not white, as opposed to Black. This is due to either the small number of incarcerated

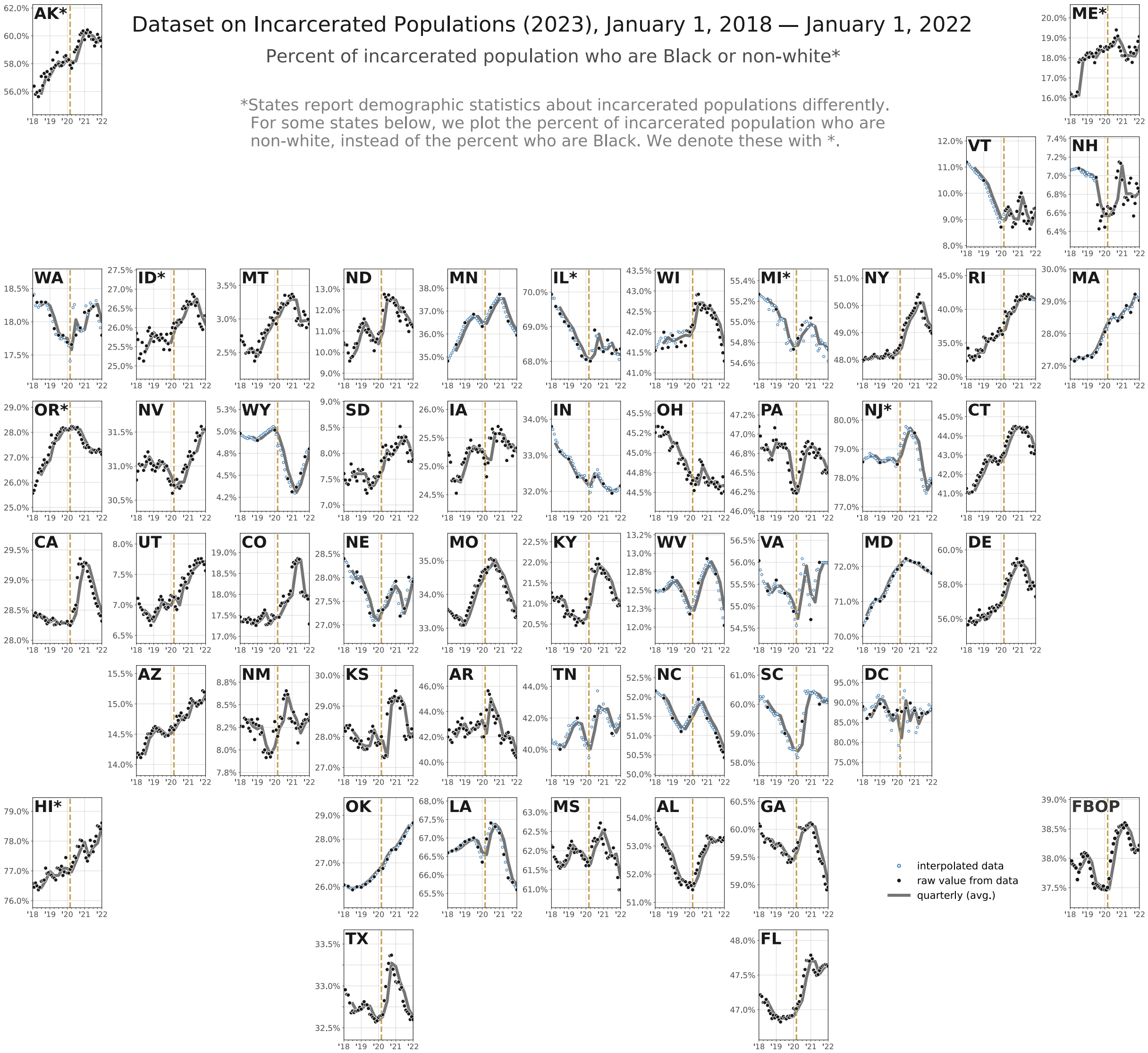

Figure A.2: State-by-state (and federal) time series of the percent incarcerated Black people over time. Note: Because of the heterogeneity in reported racial categories and some states' small incarcerated Black population, we plot the percent incarcerated population who are not white for several states. Vertical axes differ in magnitude by state. For a visualization of the states with the most standardized reporting procedures.

Black people in the state (as in Hawaii, Idaho, South Dakota, Vermont, etc.) or the absence of Black as a racial category in the state's reports (e.g. Michigan). With this state-by-state view, we see that the only state that unequivocally does not show a similar trend to Figure 1 (and in fact, shows the opposite) is Oregon; ongoing and future work will attempt to disentangle whether this is a reflection of the state's criminal justice policies, demographic patterns, reporting procedures, or any of the above.

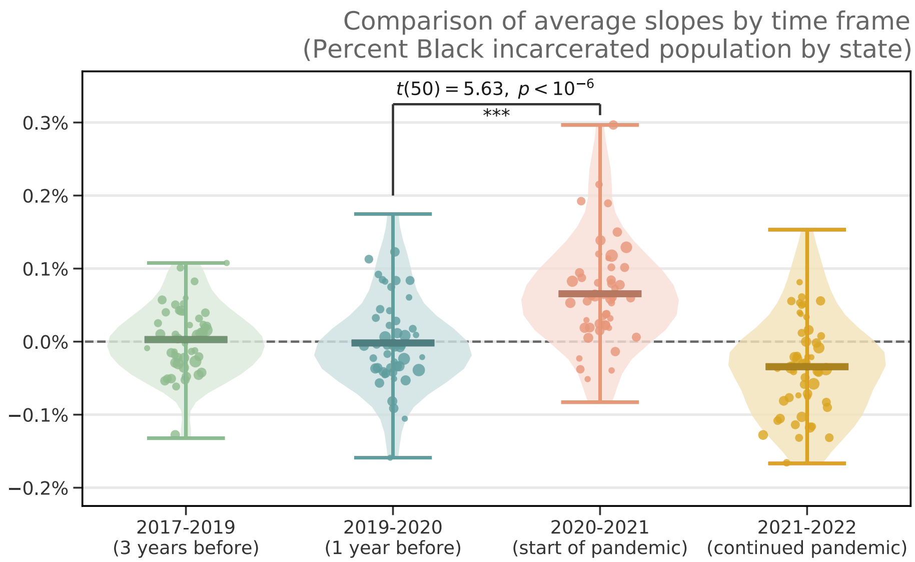

Figure A.3: Comparing slopes of percent incarcerated Black population across states. Each violin plot represents every state and federal prison system's slope of the percent incarcerated Black population curves. Violin plots depict distributions of data, such that the width of each violin corresponds to the density of points in a given range, and the lines at the top/bottom are the max/min of the underlying data. The horizontal lines in the middle of each violin plot corresponds to the means of each distribution. From 2020 to 2021, we observe a statistically significant difference in the slope from before the pandemic (two sided t-test, $t(50) = 5.6251; p = 1.691162 \times 10^{-7}$).

A.2 A nationwide trend or state-level heterogeneity?

In this section, we report raw and interpolated data for each state, the District of Columbia, and the Federal Bureau of Prisons, in order to compare time series of the percent Black population in prisons to the percent non-white population (see [4]). In some cases, the main effect observed in Figure 1B is clearly recapitulated; in others, we do not see the effect in the percent Black population, but rather we see it in the percent non-white population. The only states where we do not observe the main effect in both demographic categories are Maine, Oregon, and Wyoming.

In Figure A.3, we plot the distribution of the slopes of each state's percent incarcerated Black population data during different time windows (2017-2019, 2019-2020, 2020-2021, and 2021-2022). With few exceptions, this plot shows that the slope of the percent Black incarcerated curves increased during 2020, compared to the 2019 averages. After this, the same slopes decreased during 2021. Isolating these slopes is useful again for nationwide comparison for two reasons: 1) because it can allow us to ask questions about what we would expect to happen if we did not observe some sort of spike in the percent Black population in 2020-2021, and 2) because we can use the slope of 2019-2020 as a way of normalizing the entire time series, allowing for a simple test of whether or not we observe a spike in 2020.

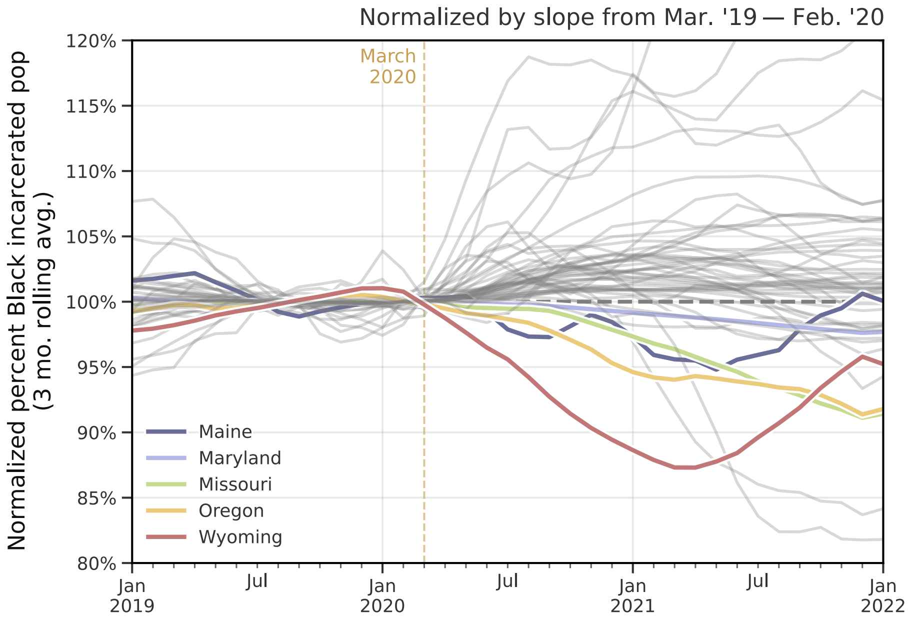

Figure A.4: Normalized percent of incarcerated Black population for every state and federal prison system. Applying the same normalization procedure as in Figure 3D (by slope of pre-pandemic curve), we compare the trends of the every state and federal prison system's percent of incarcerated Black population. Under this view, five states do not follow the broad nationwide trend as in Figure 1B: Maine, Maryland, Missouri, Oregon, and Wyoming. For every other state, we see the percent of incarcerated Black people increase for multiple months, starting after March 2020. The top four curves on the plot correspond to prison systems that are smaller in size (New Hampshire, North Dakota, Vermont, the District of Columbia), with demographic statistics that can be disrupted by small fluctuations in incarcerated populations.

Figure A.4 normalizes each state's percent incarcerated Black population data by the best-fit line from its *slope* between 2019-2020. That is, we divide each state's time series of percent Black population by the corresponding value in the best-fit line from the slope from the year prior to March 2020. Visually, this bundles the states' curves to the 100% value before the pandemic (i.e., 100% of normalized pre-pandemic values). After March 2020, there are five states that do not at least briefly show a spike in their percent Black population: Maine, Maryland, Missouri, Oregon, and Wyoming.

A.2.1 Interpolating monthly population data

In [4], we have included tables of the population and demographic data for each prison system studied here. Also included in these tables is a tag about whether or not the data is raw data from the state or interpolated. For most (35) prison systems, we have raw monthly data for the entire duration of the study period. For some, we only have data at

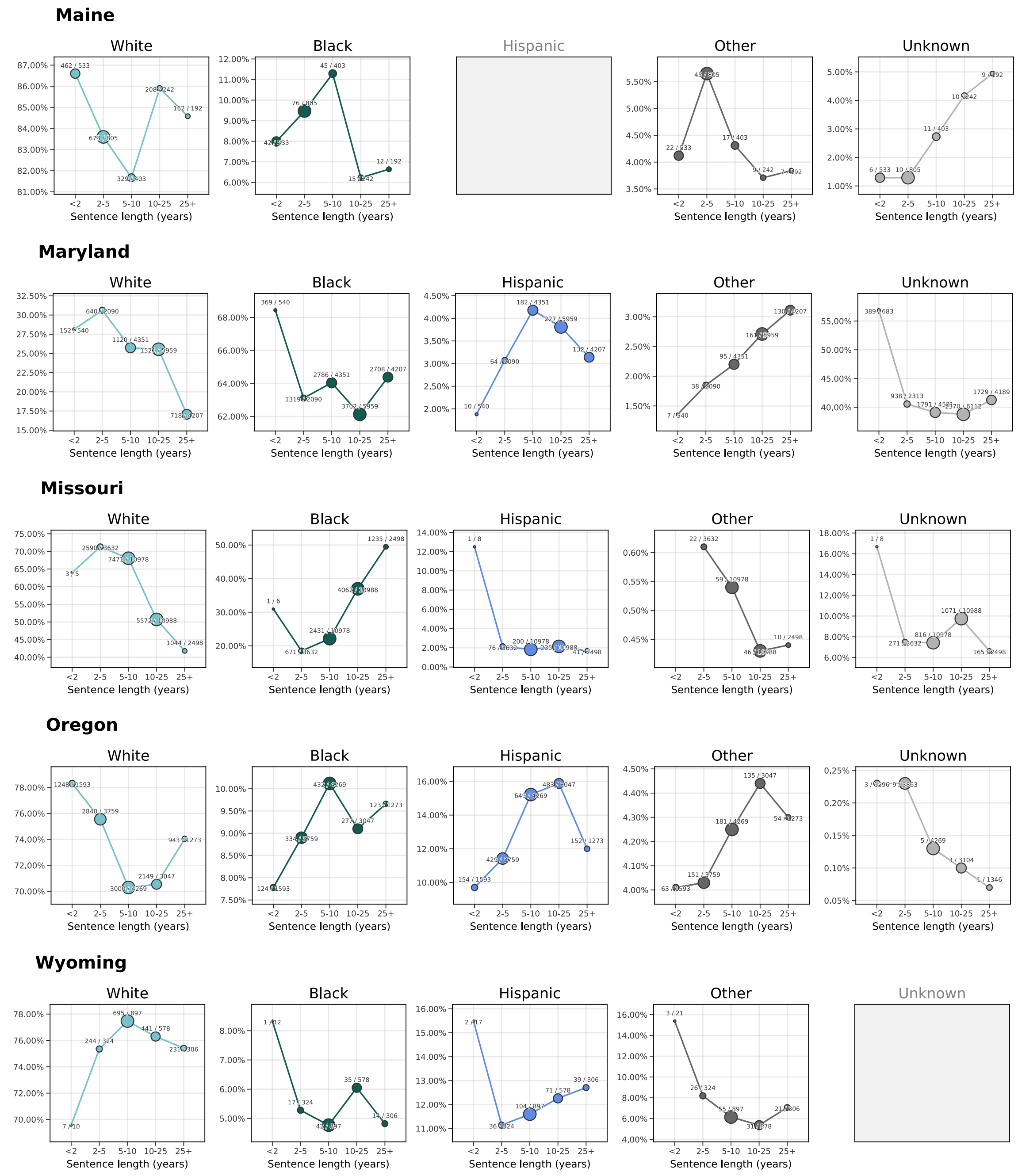

Figure A.5: Examination of sentencing patterns in the five states that do not follow the nationwide trend from Figure 1B. Data are from the NCRP [1].

the quarterly (8) or bi-annually (4); for these 12 states, we simply do a linear interpolation on the raw demographic data and sum these columns together to arrive at a total estimate for the number of people incarcerated each month in between the quarterly/biannual dates.

As a validation, we do this same interpolation on states where we do have reliable monthly

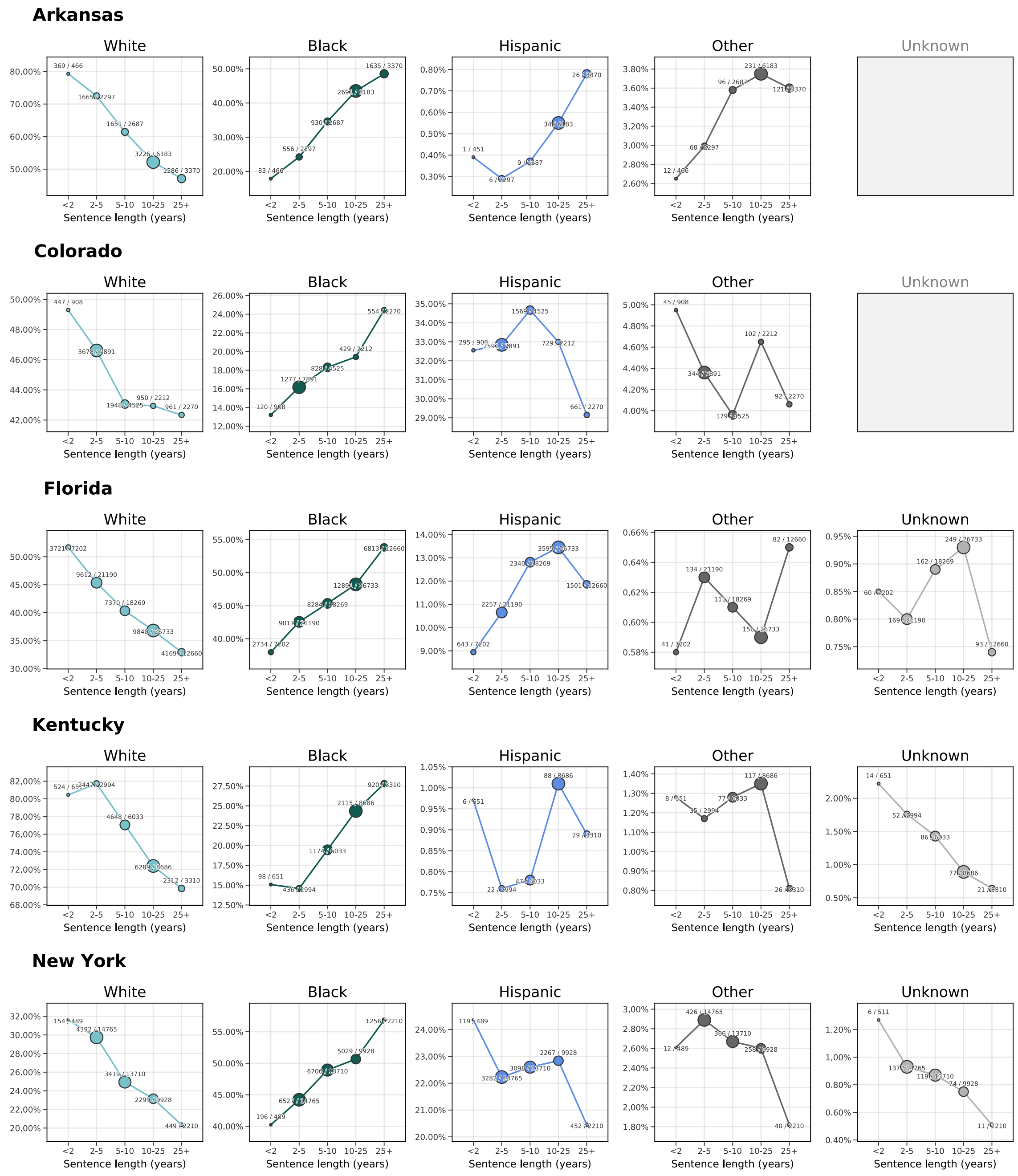

Figure A.6: Companion figure to Figure A.5, examining sentencing patterns across five states that do follow the nationwide trend from Figure 1B. Data are from the NCRP [1].

data, and the estimates are almost perfectly aligned (average R^2 of correlation with ground truth for bi-annual interpolation: 0.975; annual interpolation: 0.945). We show this high correspondence for three example states in Figures A.11, A.12, and A.13. For five states:

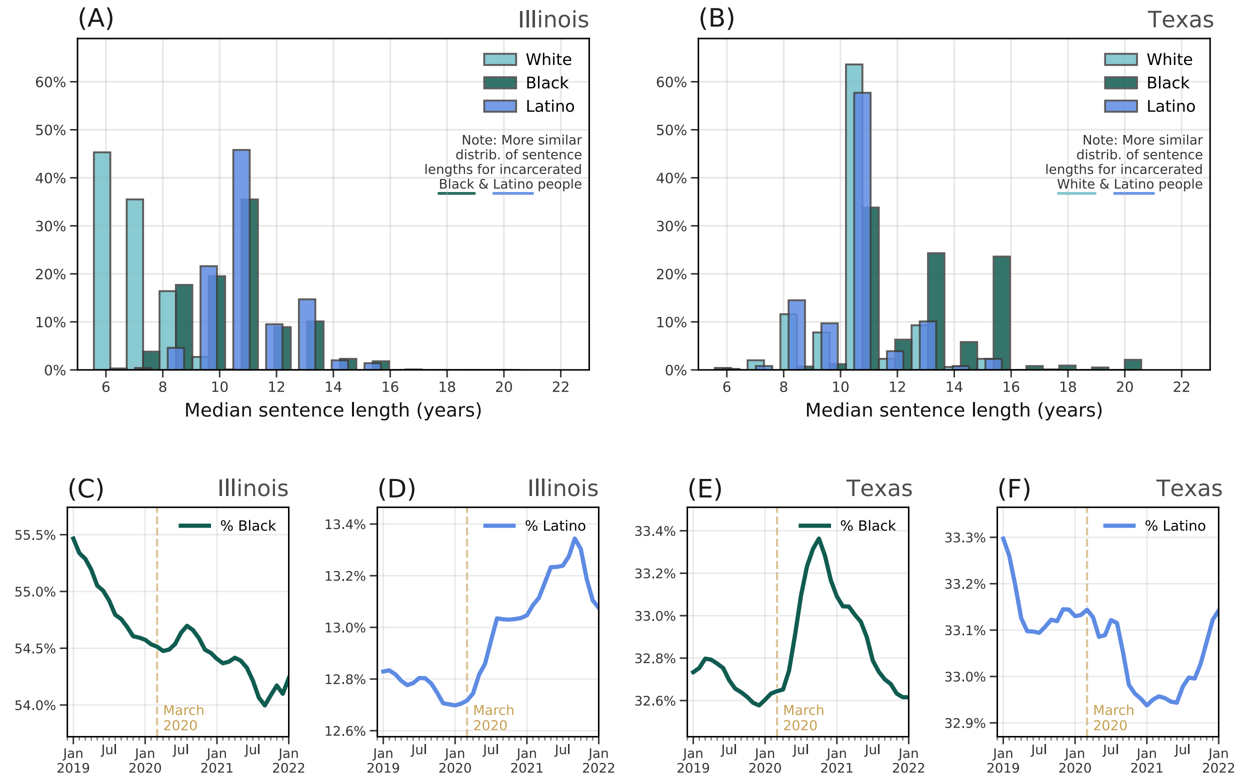

Figure A.7: Comparison of median length of sentence among Black, White, and Latino incarcerated populations in Illinois and Texas. We randomly sample 100 incarcerated White, Black, and Latino people and report the median length of sentence; by repeating this process thousands of times, we can approximate the median length of sentence, by race in **(A)** Illinois and **(B)** Texas. For each state, we compare the percent of incarcerated people who are Black **(C & E)** and Latino **(D & F)**, highlighting the importance of racial differences in sentence length in driving the trends we observe in this study.

Michigan, New Jersey, South Carolina, Tennessee, and Virginia, we only have demographic data at the yearly level. In each of these states, we have population totals at the monthly level; with these, the task becomes to estimate the counts of incarcerated people by race each month, given the population totals. In some ways, this is an easier task, since we know the overall trend in the prison population. Here, again, we do a linear interpolation between the dates without missing values, multiplied by a factor of (interpolated_sum / actual_sum). Doing this same validation on states with reliable, monthly data reporting gives us high alignment again. Lastly, we note that every combination of including or excluding states based on their reporting frequency and quality still produce the same qualitative results, which we would expect given the extensive discussion above.

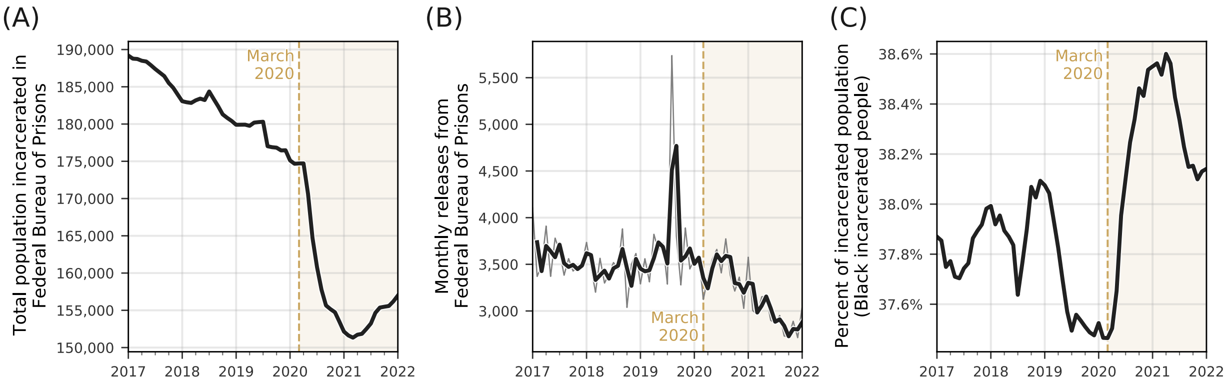

Figure A.8: Federal Bureau of Prisons population data. **(A)** Total number of people incarcerated in the Federal Bureau of Prisons (FBOP) (monthly, from January 2017 until January 2022). **(B)** Total number of people released from FBOP (lighter curve: monthly; darker curve: two-month rolling average). During the pandemic, releases decreased alongside decreases in the total population; notably, we do not see months with unusually high numbers of releases once the pandemic starts. **(C)** Percent of total FBOP population who are Black.

A.3 Comparison across prison population datasets

Other organizations collect and report data about prison populations over time. In order to situate the data used here within a broader body of work studying U.S. prison population trends, we validate against data released by the Bureau of Justice Statistics [2] (BJS) and the Vera Institute for Justice [3] (Vera). In Figure A.10, we plot the BJS's yearly estimates of the number of people in state prisons across the United States from 2014 until 2020. We concatenate the BJS data with the Vera data to approximate a "ground truth" estimate for the prison population over time.

We note several key points. First, starting in 2020, our dataset almost exactly matches the Vera dataset. Prior to 2020, our dataset reports a prison population that is approximately 1% smaller than the BJS data. After investigating what could have brought about these differences between the two datasets, we identified five states with the largest between-dataset differences (Montana, Florida, Texas, Virginia, and Ohio; see Table A.3). Because of these discrepancies, we took additional care to confirm that the data we had collected was exactly what was reported by states' Departments of Corrections websites (or sometimes, through Freedom of Information Act requests). In Section A.3.1, we describe the rationale for why we are confident in the data included in the present study, and we also directly link to the data sources used to offer transparency in the data collection process.

A.3.1 Comparison to Bureau of Justice Statistics data

To our knowledge, the scale of the data that we assembled in this work is unique among the available public datasets about states' prison populations over time. In Table A.3, we dive deeper into the discrepancies between the data used here and those that were released by the

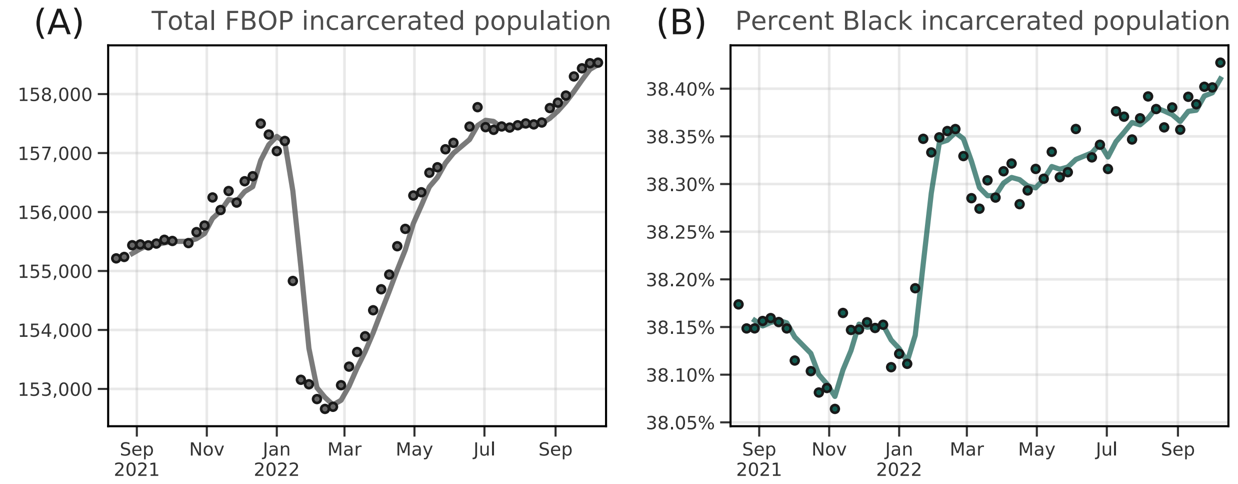

Figure A.9: Case study: Releases and race in FBOP populations. (A) Total number of people incarcerated in the Federal Bureau of Prisons (FBOP) (weekly, from September 2021 until September 2022). **(B)** Percent of total incarcerated population who are Black.

Bureau of Justice Statistics [2]. We offer explanations that reconcile why we may observe such differences, and we conclude that the data reported here is consistent with state prison population statistics reported by states' Department of Corrections.

A.3.2 Comparison with the National Corrections Reporting Program

We drew on another large, well-known dataset to validate the findings from the data we collected: the National Corrections Reporting Program (NCRP) [1]. The NCRP data we used contains detailed information about individuals incarcerated in almost every state, yearly, until December 31, 2019. In recent years, data from the NCRP has been subject to scrutiny both in terms of its coverage and completeness as well as how it reports data about the race of incarcerated people [5–9].

Nevertheless, these data are a cornerstone of legal and justice research in the United States, and as such, we sought to use it as a benchmark of pre-2020 data to see 1) the extent to which there is significant overlap between the two datasets and 2) if we analyzed the subset of our data that only had high overlap with the NCRP data (i.e., exclude states where there is significant disagreement between the two datasets), whether we would reproduce the main result in Figure 1B.

In Figure A.14, we plot a comparison between the NCRP data and our data for 12 states' total incarcerated population over time (NCRP data plotted in red). Immediately, we are reassured about the correspondence between these states in the two datasets. There are, however, several states where the overall trend in the prison population is similar between the two datasets, just shifted uniformly up or down (i.e. states with the same or similar-shaped curves but shifted by a fixed amount). Lastly, there are several states where the NCRP data is clearly not capturing the same information that our dataset contains. These

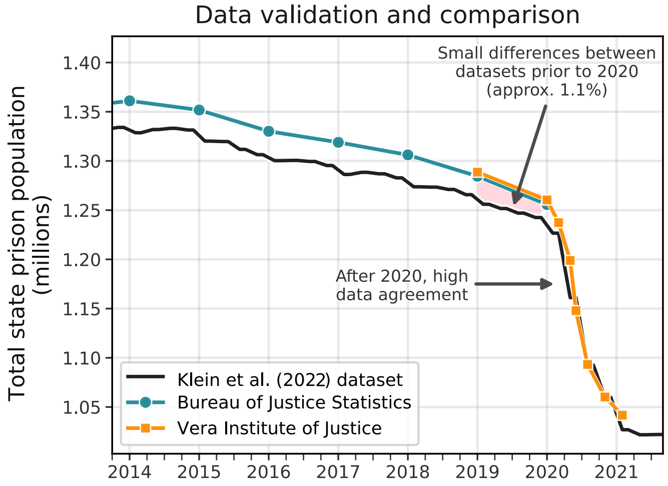

Figure A.10: Data validation. We compare the novel data presented here to data from the Bureau of Justice Statistics [2] and the Vera Institute for Justice [3], finding high data agreement during 2020 and early 2021. There are small differences between our dataset and the Bureau of Justice Statistics prior to 2020, but see the Data & Methods section and Table A.3 for further explanation of these differences.

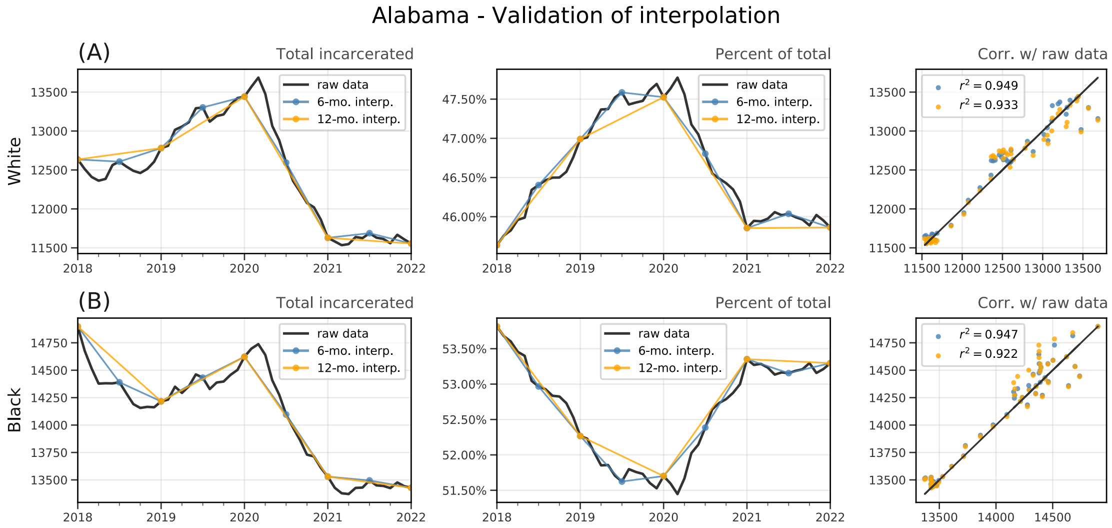

Figure A.11: Validation of interpolation on high-quality data states: Alabama.

are states that—we suggest—do not have high coverage or high data quality in the NCRP dataset or have otherwise changed their reporting protocol during the duration of their inclusion in the NCRP.

Ultimately, if we only analyze states with high overlap between NCRP and our data (a proxy for overall reporting quality: Washington, California, Nevada, Utah, Nebraska, Arizona, Colorado, Wyoming, Kansas, Texas, Iowa, Minnesota, Illinois, Indiana, Kentucky,

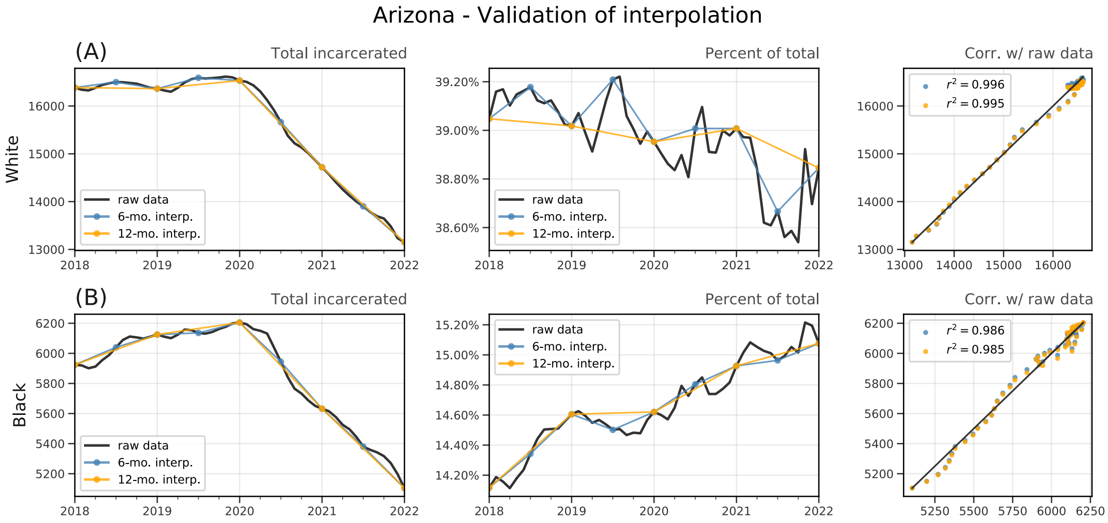

Figure A.12: Validation of interpolation on high-quality data states: Arizona.

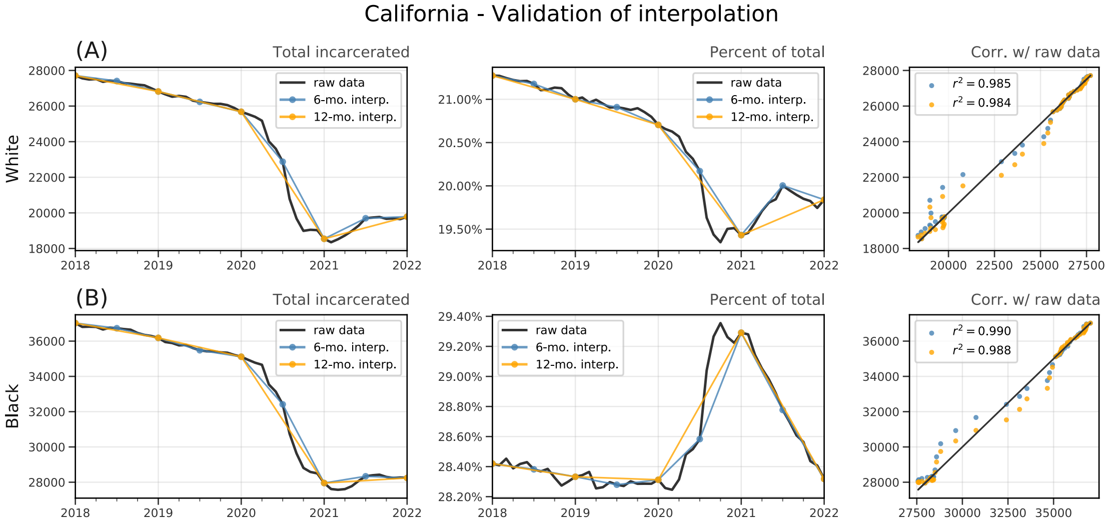

Figure A.13: Validation of interpolation on high-quality data states: California.

Tennessee, Mississippi, West Virginia, Ohio, Wisconsin, Georgia, Florida, New Jersey, New York, and South Carolina), we see the same qualitative result (see Figure A.15). We see this as a validation with multiple benefits: First, it grounds the data we have collected in a known to a well-studied companion dataset. Second, we see an opportunity to use our data to augment or help fill in states with known reporting irregularities or other issues in the NCRP dataset. While the insights from these comparisons between the two datasets were not the intended contribution of our paper, we are encouraged nonetheless that these analyses offer a roadmap for future work improving data quality in the NCRP.

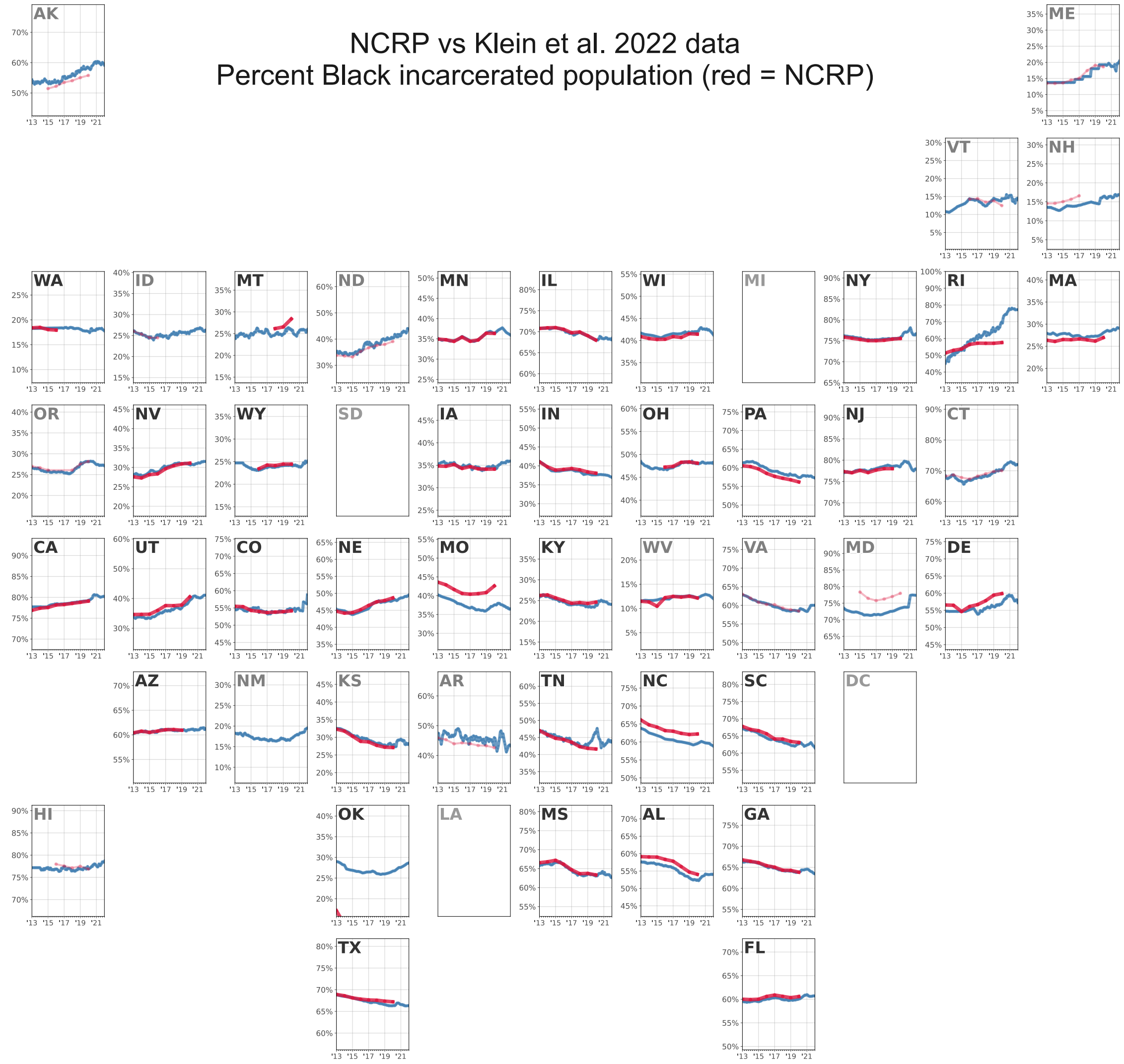

Figure A.14: Comparison of Klein et al. dataset to historic data from the National Corrections Reporting Program data. States with light grey labels (e.g. Alaska, Oregon, Hawaii, Idaho, etc.) are not included in the "term file" (states categorized as having more reliable data). Washington, California, Nevada, Utah, Nebraska, Arizona, Colorado, Wyoming, Kansas, Texas, Iowa, Minnesota, Illinois, Indiana, Kentucky, Tennessee, Mississippi, West Virginia, Ohio, Wisconsin, Georgia, Florida, New Jersey, New York, and South Carolina are the states with the highest degree of correspondence between the two datasets.

A.3.3 Survey of states' race reporting procedure

As briefly mentioned in the previous section, there remain several known challenges in relying on administrative data to study racial disparities. In particular, one common issue is the heterogeneity between how states and other government agencies report data on race. Broadly, there are two main approaches to collecting information about an individual's race and/or

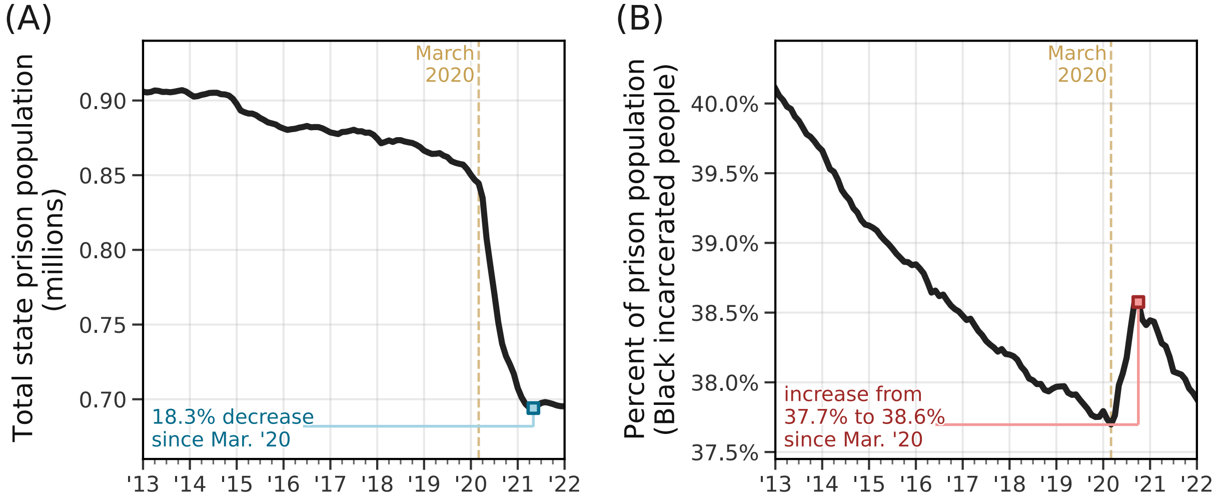

Figure A.15: Replication of Figure 1, based only on data from states with high overlap with NCRP data. If we reproduce our main result using only states with high overlap between the NCRP data and our own (a proxy for overall reporting quality: Washington, California, Nevada, Utah, Nebraska, Arizona, Colorado, Wyoming, Kansas, Texas, Iowa, Minnesota, Illinois, Indiana, Kentucky, Tennessee, Mississippi, West Virginia, Ohio, Wisconsin, Georgia, Florida, New Jersey, New York, and South Carolina), we see the same qualitative result.

ethnicity: self-reported from the individual in question or "visual inspection" by either a clerk, law enforcement official, or other administrative staff. On the one hand, some scholars argue that external assignment of race more accurately reflects the scale at which discrimination or prejudice operates [10] and should therefore be relied upon for studying race and ethnicity. On the other hand, self-reported data about an individual's race is likely more accurate and therefore more useful for large-scale quantification [11, 12]. Many researchers argue for an array of different survey questions in order to accommodate both approaches [13, 14], while others have found ways of improving the accuracy of administrative race data through a variety of post hoc statistical corrections [15, 16]. An Oregon Criminal Justice Commission report, for example, analyzed race/ethnicity data in their management system and discovered that the system regularly mis-labeled individuals' race, such Latino, American Indian, and Asian individuals were under-represented by up to 15% [17]—a number that can be corrected through statistical re-weighting. Table 5 of the BJS *Prisoners in 2020* report emphasizes this issue and describes the steps taken to statistically adjust the data in order to estimate the underlying racial distribution in state and federal prisons [18]:

> "National-level estimates of the number of persons by race and ethnicity under the jurisdiction of state prisons on December 31, 2020 were based on an adjustment of NPS [National Prisoner Statistics] counts to comply with the Office of Management and Budget (OMB) definitions of race and ethnicity... Not all NPS

> providers' information systems categorize race and ethnicity in this way. In addition, these data are administrative in nature and may not reflect a prisoner's self-identification of race and ethnicity... For state prisoners, BJS calculated the ratio of the distribution of state prisoners by race and ethnicity in BJS's self-reported prisoner surveys, which use OMB categories for race and ethnicity, to the distribution of prisoners by race and ethnicity in NPS data for the year closest to the fielding of the survey. BJS then multiplied this ratio by the distribution of state prisoners' race and ethnicity using the current year's NPS. The percentage of persons self-reporting to the NPS as non-Hispanic and as two or more races was assumed to be equal to that of the self-reported prisoner survey. The final percentage distribution of race and ethnicity was multiplied by the total of sentenced state prisoners to obtain counts for each category."

In other words, the BJS performs a statistical correction that attempts to correct for heterogeneity in how race is reported across states. A natural question that arises here is whether we think that the same heterogeneities in administrative race reporting are present in the dataset we introduce here. If there were systematic differences in how race is reported by state (e.g. some states may report the race of a newly incarcerated person via a self-reporting procedure during intake; other states may record this data via a staff member assigning a race during intake based on visual features), this could potentially be problematic for the main results in this work. That is, we may be systematically mis-estimating the magnitude of the observed trends in Figure 1B. Alternatively, it may be even more problematic if there are non-systematic differences in how race is reported; in this case, it could potentially require a different statistical correction performed for each state, opening the dataset up to a deserved scrutiny. Fortunately, we do not think the dataset used in this work is subject to these concerns—or if it is, the impact is minimal. The reason for this is based on newly collected data from almost every state's Department of Correction, displayed in Table A.4[1].

In nearly every state, it is the stated policy to collect self-reported race data during admissions into prison. There are a few states with either ambiguous policy language (e.g. Minnesota, which explicitly writes "Race information may be self-identified or classified by an observer.") or policy language that is suggestive of self-report but not entirely (e.g. Massachusetts: "It is mostly self-reported, however, if the county sends a face sheet the Booking Officer will use that."). One state (Texas) explicitly referred to staff members visually assigning someone's race "...during intake, the [Texas Department of Criminal Justice] will visually determine the race of the individual." While Texas was the only state that uses this procedure for collecting race statistics, we must stress that it is the largest prison system in the country, and trends in data from Texas strongly influence national averages.

The other states' policies are based on self-reported data; we include the precise language from the policy in Table A.4. We do not know of other research with this kind of detailed state-by-state data. Note: here, we do not assume perfect compliance with the self-reporting policy (e.g. either on the administrators or the people being admitted into prison), but at

[1] Table A.4 is still awaiting answers from Washington D.C., Alabama, and Louisiana.

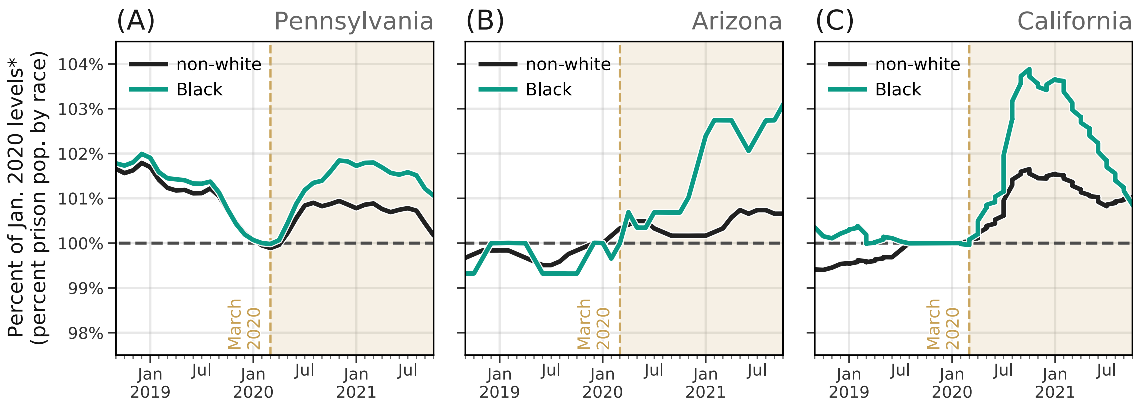

Figure A.16: Larger effect among incarcerated Black people. Comparison of the relative increase in the proportion of incarcerated people who are non-white vs. black in three states during the pandemic. This trend is especially pronounced in the three states above, however there are several states where the opposite is true. Further distinguishing these effects will be the subject of future research.

this point, we do see this survey of states' race data collection policies as the most promising validation of the administrative race data used in this work.

A.4 Case studies: Court system, policing, and inmate release data

In addition to data about states' prison populations and prison policy, we also used state-specific data about outcomes of court proceedings, crime/offense type and severity, traffic stops, and inmate releases in order to tell a broader story about the structural effects of mass incarceration during the COVID-19 pandemic.

A.4.1 Florida trial statistics data

Using Florida as a case study, we show how changes to typical court proceedings can potentially lead to new racial disparities in the prison population [19–22]. In Figure A.18, we plot monthly trial statistics from circuit criminal defendants in Florida. Prior to March 2020, an average of 14,000 defendants were disposed each month (i.e., pass through the court system and have their charges dropped, agree to a guilty plea, go to a jury trial, or go to a non-jury trial; Figure A.18A). Starting in March 2020, the number of defendants decreased sharply, reaching nearly 4,000 in May 2020, resulting in a backlog of cases (Figure A.18B).

Between March 2020 and June 2020, more than 99% of cases did not go to trial (up from an average of approximately 97% prior to 2020). An increase in the proportion of cases that get resolved pre-trial means that a greater percent of all defendants passing through the Florida courts system will either agree to a guilty plea or see their charges dropped

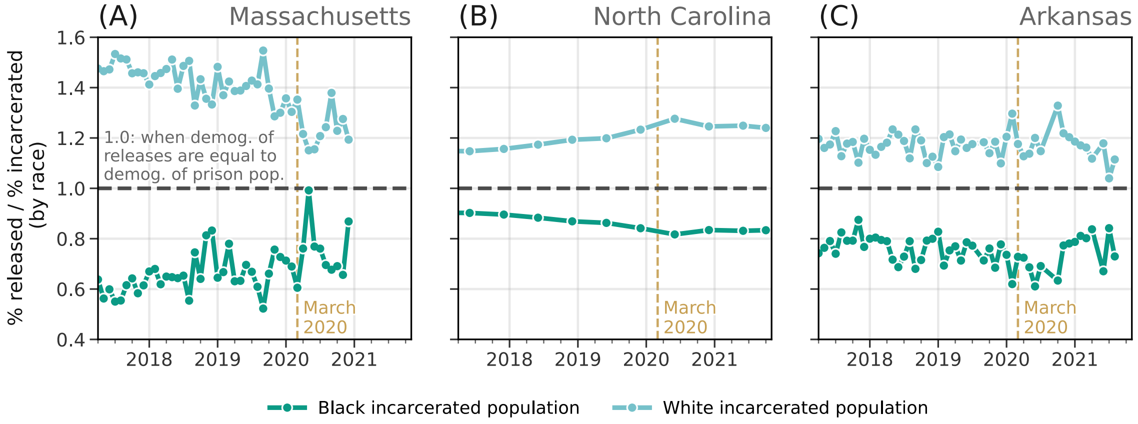

Figure A.17: Ratio of race of releases to race of prison population. Here, we highlight the evolution of the ratio between r_x/N_x, where r_x is the percent of releases who are race x and N_x is the percent of the incarcerated population who are race x (in this case, we use Black and white incarcerated people for x). If this ratio is 1.0, there is a proportional number of releases as one would expect, given the demographic composition of the prison population. In the three states included here, white incarcerated people account for a larger share of releases than one would expect, given the demographic distribution of the prison population; conversely, incarcerated Black people are released at lower-than-expected rates.

entirely. Importantly, both of these can be sources of statistical bias in the resulting prison population.

First, prior research has demonstrated that Black defendants are almost 70% more likely than white defendants to receive a plea deal that involves spending time in prison [23]. Second, we show here that the percent of cases that were dismissed entirely increased from an average of 10% before 2020 up to 15% in June 2020 (Figure A.18C), and this increase in dismissed cases is strongly correlated with the percent of non-white incarcerated individuals (lagged one month to account for time delays in sentencing; Figure A.18D). This correlation did not simply arise after March 2020—we see these same correlations between percent of dismissed cases and percent of non-white incarcerated people prior to the COVID-19 pandemic, suggesting that this relationship is potentially more general and not merely an anomaly due to the pandemic. This case study highlights multiple potential disparities that can stem from disruptions in the court system, and it emphasizes the need for more states to make similar sentencing data available to the public. In this vein, we report preliminary analyses about the race of defendants whose cases were dismissed in Figure A.19, using data compiled by the Florida Office of the State Courts Administrator (collected by court clerks) to show a relative increase in the number of white defendants among those who had cases dismissed in the early months of the pandemic. See Section A.4.1 for further discussion of these data, including limitations and future work. Having access to standardized data about the race of defendants across every state will further allow us to connect case-dismissals to prison demographic distributions.

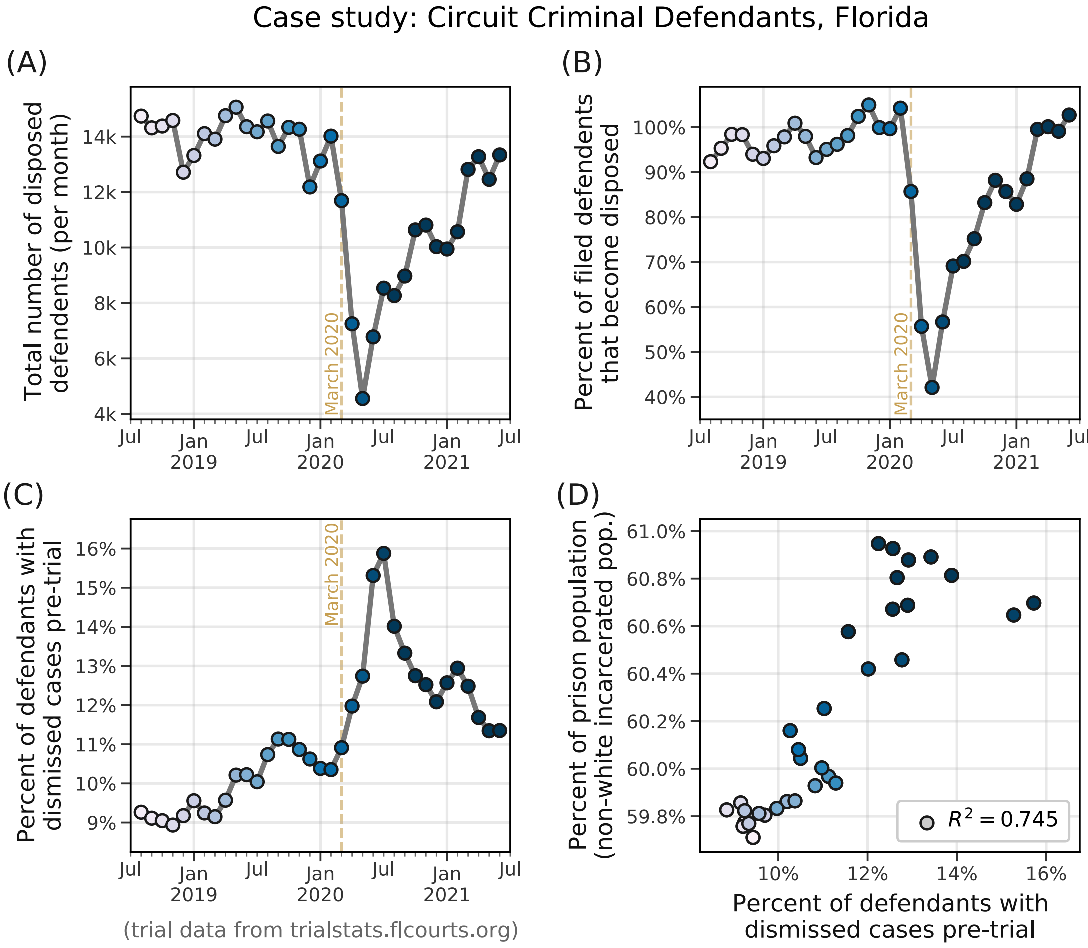

Figure A.18: Circuit criminal defendants in Florida. (A) Total number of defendants with disposed cases (i.e., number of closed cases) over time. **(B)** Case completion rate (number of disposed defendants divided by the number of cases filed). **(C)** Percent of defendants with cases that are dismissed before going to trial (different from pre-trial guilty pleas). See Figure A.19 for preliminary data about the demographics of defendants with dismissed cases—namely, that there is a relative increase in white defendants with dismissed cases during this period. **(D)** Correlation between percent of dismissed cases and percent of incarcerated individuals who are non-white (one month lag). Data from Florida Office of the State Courts Administrator [19], from July 30, 2018 to June 30, 2021 (latest data available).

To collect the court proceedings data used in Section 2.3 [19], we manually downloaded monthly data about the statewide data on the outcomes of Circuit Criminal Defendants between January 2018 and June 2021. We summarize this dataset in Table A.5.

In addition to summarized monthly statistics about the nature and outcomes of circuit criminal court defendants, we also requested *individual*-level data about the defendants that make up this aggregate data. The Office of State Court Administrator (OSCA) in Florida

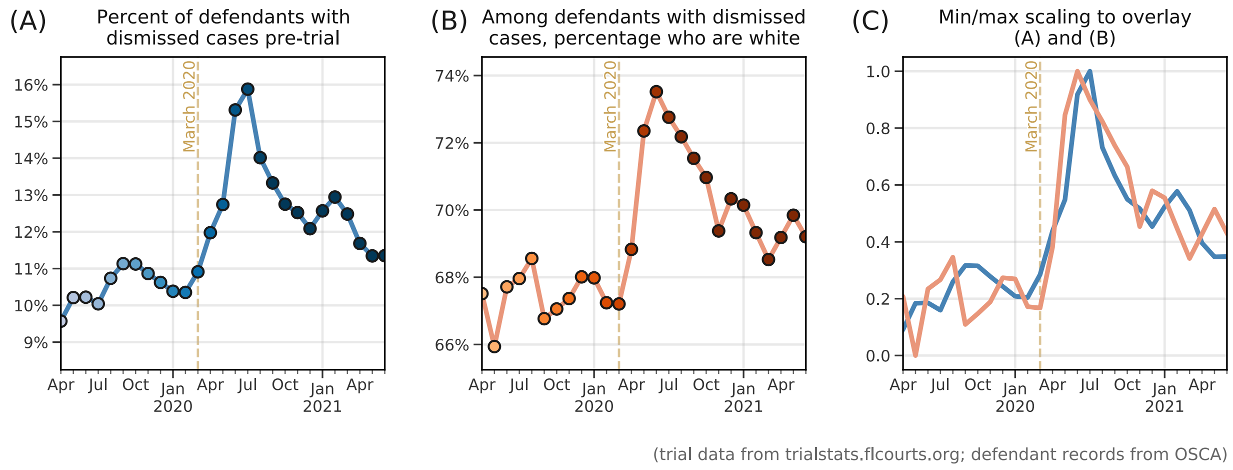

Figure A.19: Florida pre-trial court dismissals, by race. (A) Reproduction of Figure A.18C. **(B)** Using data compiled by the Florida Office of the State Courts Administrator (OSCA) from the Criminal Transaction System (CTS), we can begin to get a sense of the demographics of the defendants with dismissed cases (Note: OSCA stresses that these data are provided by court clerks and are subject to change, and that the conclusions and analysis that is done using this data are solely those of the authors of this paper). **(C)** Comparison of the curves in **(A)** and **(B)** via min-max scaling.

provided us with data from the Criminal Transaction System for 2018, 2019, 2020, and 2021; these data contain a column for defendants' race, the action taken by the court, and the date each case was decided (among many other variables)[2]. This dataset allows us to run a simple analysis: Among defendants with cases that were dismissed, between 2018-2021, what percent are recorded as white? We plot this in Figure A.19B (A.19A is a reproduction of Figure A.18C, and A.19C shows the two curves atop one another, rescaled using min-max scaling in order to highlight the timing and relative increase that both measures show after March 2020).

A.4.2 Texas offense severity data

In recent years, White/Black/Latino people have accounted a for similar percent of the total incarcerated population in Texas state prisons (33.7%, 32.6%, 33.1%, respectively); mirroring the nationwide trend in Figure 1B, the percent of incarcerated Black people increased sharply in Texas after March 2020 (Figure A.20A). While it is difficult to point to any single cause behind this abrupt disparity, some have argued that more careful consideration must be given to racial differences in the severity of the crimes for which people are incarcerated.

[2]Note: OSCA is the organization that compiled the data and are not the custodians of the data. Clerks of the court record each defendant's case data via the Offender Based Transaction System. Any conclusions or analysis that will derive from this dataset are solely those of the individual author(s) or the person(s) who did the analysis and not of the Florida Office of State Court Administrator.

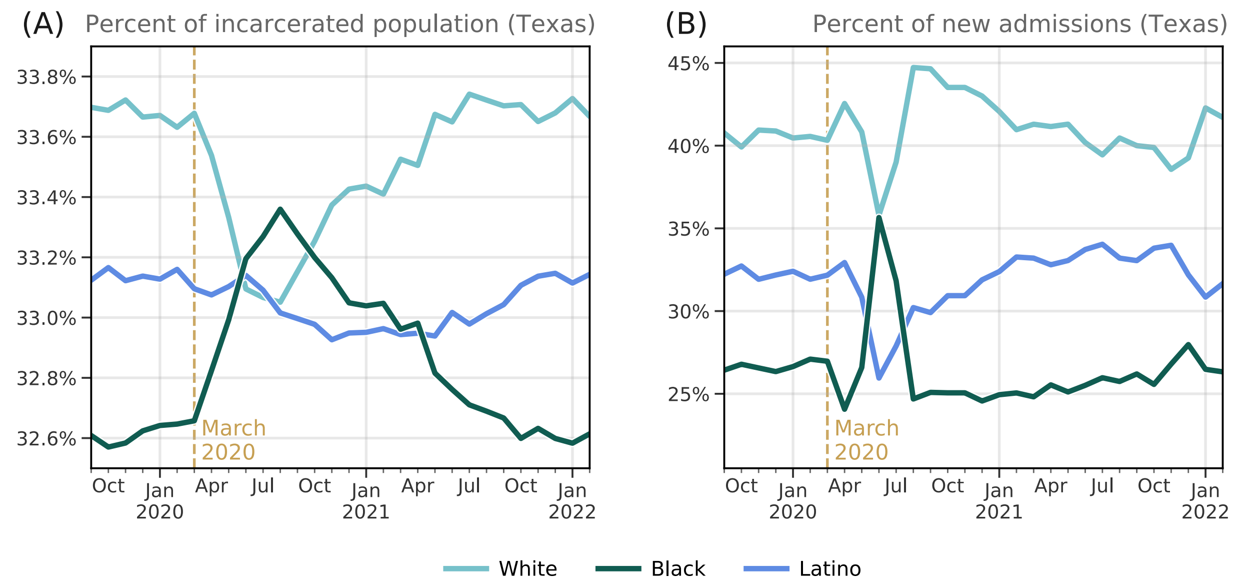

Figure A.20: Race of incarcerated population and monthly admissions in Texas. **(A)** Incarcerated population in Texas over time, by race. **(B)** Monthly percentage of new admissions into Texas state prisons, by race.

That is, without more extensive data about the incarcerated population in Texas, we cannot rule out the possibility that the observed spike in the relative number of incarcerated Black people is due to a relative increase in the severity of crimes committed, by race. We show, however, that this is not the case.

To do this, we merge data from two sources. The first is monthly data about every incarcerated person in Texas from July 2019 until November 2021 (the Texas Department of Criminal Justice "High Value Dataset" series, from `https://www.tdcj.texas.gov/kss_inside.html`); each row in this dataset corresponds to an incarcerated person and includes details about the individual's race and sex, as well as sentencing information. The second dataset is a table that maps every offense to one of four severity levels: low, moderate, high, or highest (`https://www.tdcj.texas.gov/bpp/parole_guidelines/Offense_Severity_Class.pdf`).

We assign an offense-severity category to each incarcerated person by merging the two datasets along the National Crime Information Center (NCIC) code for the sentenced offense. In Figure A.21, we compare these offense-severity categories by race. In Figure A.21A-D, we plot the race distribution within each offense-severity category (i.e., we plot the percent of incarcerated persons with a given offense-severity category who are White/Black/Latino). To accentuate the relative changes in these trends, in Figure A.21E-H, we plot the same curves standardized (i.e., divided by) by their pre-pandemic averages; in these subplots, 100% would indicate no difference from before the pandemic. After March 2020, we see abrupt increases in the relative number of incarcerated Black people in the "low" and "moderate" offense-severity groups (dark green curve, Figure A.21E-F). This is exactly counter to the suggestion that

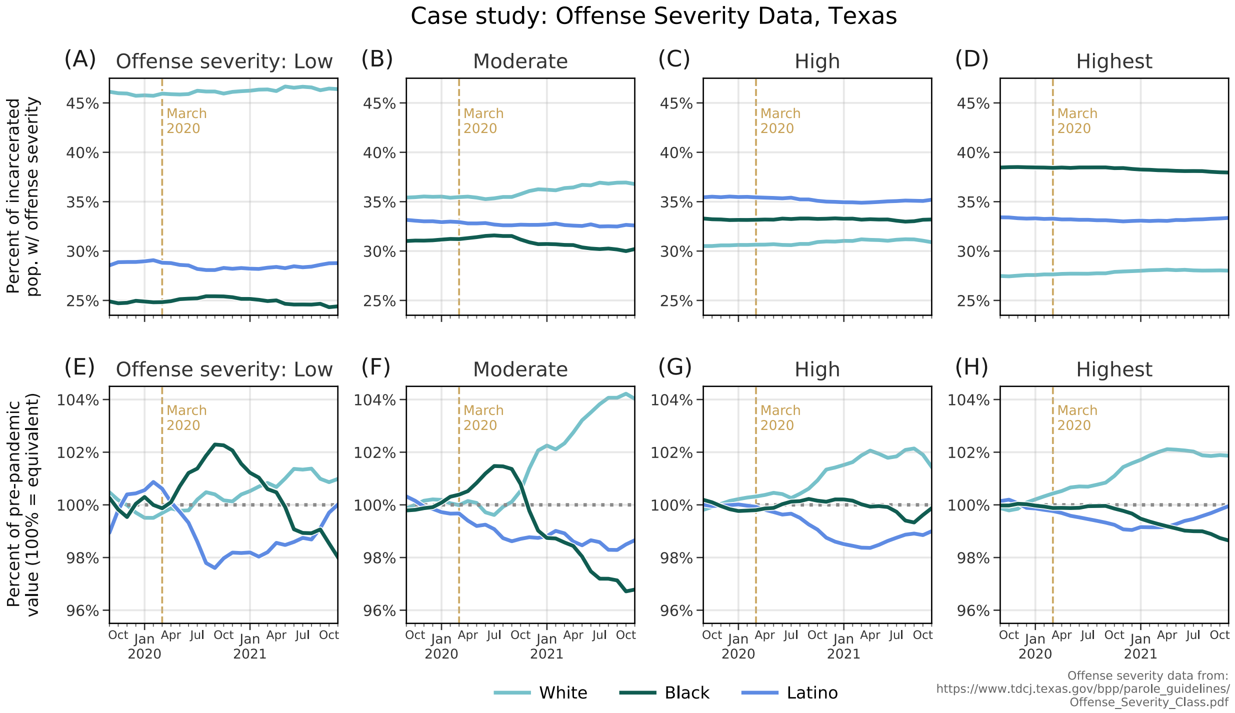

Figure A.21: Offense severity of incarcerated persons in Texas, by race. Top row (A-D): For each offense-severity category (low, moderate, high, and highest), we plot monthly time series of the percent of each group who are White, Black, or Latino incarcerated people. Bottom row (E-H): The same curves as A-D, standardized by each race group's average values prior to March 2020. A value of 100% indicates no change relative to pre-pandemic averages.

the nationwide trends in Figure 1B are due to Black people committing more severe crimes during the early months of the pandemic. Without these same datasets for every state, we cannot yet say that trends seen in Texas are universal across the United States, but following these analyses, we urge every state to make these types of data available.

A.4.3 Arkansas eligible release data

In Figure A.23, we compare the demographics of the prison population in Arkansas in May 2020 to the demographics of incarcerated people eligible for early release under Governor Hutchinson's authorization [24]; despite the fact that 57.2% of the Arkansas prison population was white, over 72% of the incarcerated people eligible for early releases were white—a disparity that we would not expect to see in a prison system absent of release policies that favored incarcerated white people. These outcomes may manifest in multiple ways: sentencing patterns that create longer sentences for incarcerated Black and Latino individuals, different classifications (e.g., violent or nonviolent) and other categorizations that may drive a disparity in those released.

In Figure A.17 we show that while, on average, incarcerated Black people are released at

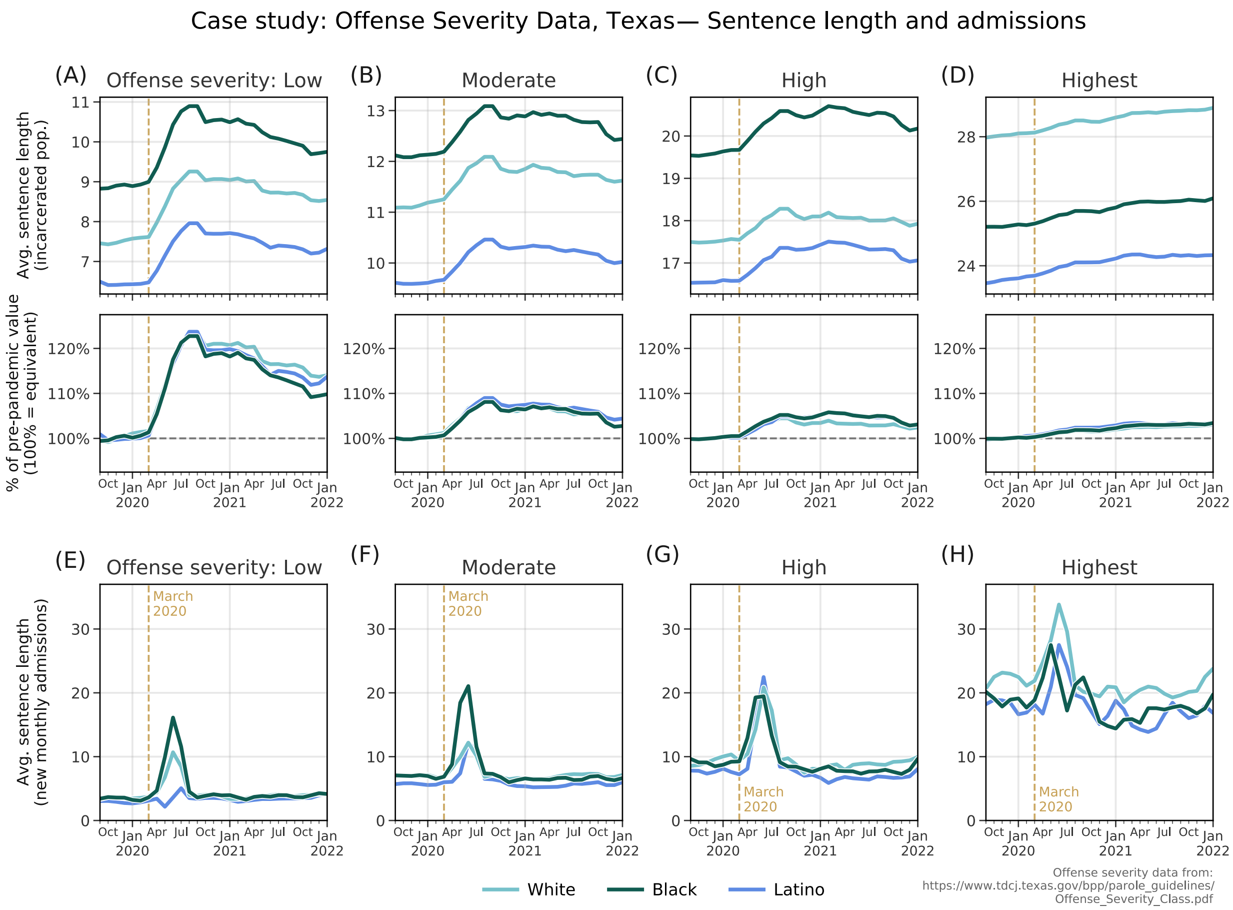

Figure A.22: Comparison of average sentence length in Texas, by race and offense severity. (A-D) Top: Average sentence length of incarcerated population in Texas state prisons (excluding life-sentences and sentences over 80 years), by race and offense severity. Bottom: Change relative to pre-pandemic values (100% indicates no change). For all offense types, we see increases in the average sentence length at the start of the pandemic due to the drop in monthly prison admissions without commensurate decreases in releases. **(E-H)** Average sentence length of new prison admissions, by offense severity and race.

disproportionately low rates (and incarcerated white people are released at disproportionately high rates), the effect of COVID-19 based releases can temporarily bring about release patterns that are less statistically skewed by race. We see this in Massachusetts especially in May and November of 2020, a sign that in at least one state, it is possible to decarcerate in a way that does not exacerbate existing racial inequalities. A key insight into why this might produce more equitable releases-to-incarcerated ratios in general has to deal with differences in the average length of prison sentence, by race (sentence length, here, we consider to be a proxy—albeit an imperfect one, see [25]—for "time served" in prison). That is, if there are systematic differences in the length of prison sentences by race (e.g. if, on average, Black people in prisons are more likely to be sentenced to longer sentences than white people, which

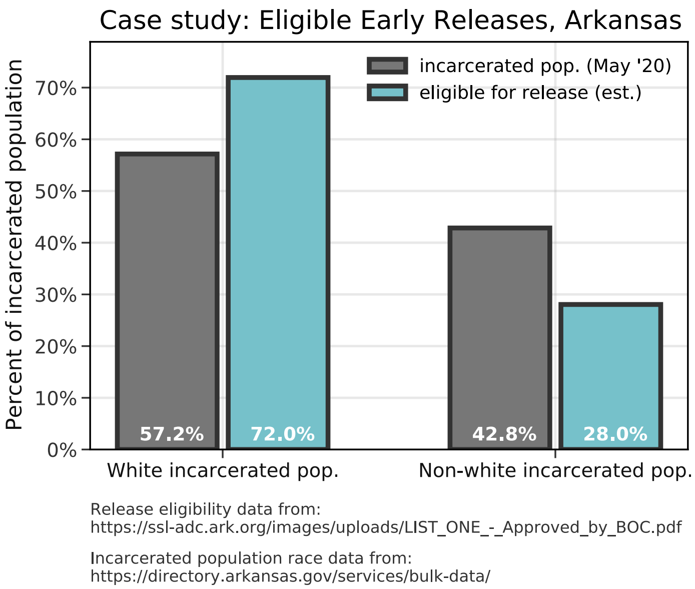

Figure A.23: Case study: Eligible releases in Arkansas. In May 2020, incarcerated white people accounted for 57.2% of the total prison population in Arkansas. However, white incarcerated people accounted for over 72% of the those listed as eligible for early release under public health precautions for COVID-19 [24].

we show in Figure A.22 for Texas), a relative increase in the number of monthly releases would decrease the overall average sentence length, provided that the release criteria is based in part on people who are close to the end of their sentence.

While there are not particularly noticeable changes after March 2020 in the trends of both curves in Figure A.17C, here we directly quantify racial disparities among the inmates who were eligible for release [24]. Using data released by the Arkansas Department of Corrections, we joined inmate identification numbers to their listed race and sex according to the Arkansas Department of Corrections Inmate Search tool (`https://apps.ark.org/inmate_info/index.php`).

A.5 Citation diversity statement

Recent work has quantified bias in citation practices across various scientific fields; namely, women and other minority scientists are often cited at a rate that is not proportional to their contributions to the field [26–33]. In this work, we aim to be proactive about the research we reference in a way that corresponds to the diversity of scholarship in this field. To evaluate gender bias in the references used here, we obtained the gender of the first/last authors of the papers cited here through either 1) the gender pronouns used to refer to them in articles or biographies or 2) if none were available, we used a database of common name-gender combinations across a variety of languages and ethnicities. By this measure (excluding citations to datasets/organizations, citations included in this section, and self-citations to the

first/last authors of this manuscript), our references contain 28% woman(first)-woman(last), 22% woman-man, 12% man-woman, 12% man-man, 0% nonbinary, 12% man solo-author, and 14% woman solo-author. This method is limited in that an author's pronouns may not be consistent across time or environment, and no database of common name-gender pairings is complete or fully accurate.

Supplemental References

[1] United States Bureau of Justice Statistics. *National Corrections Reporting Program, 1991-2019: Selected Variables*. 2021. DOI: 10.3886/ICPSR38048.v1.

[2] E. Ann Carson. "Prisoners in 2019". In: *Bureau of Justice Statistics* 255115 (2020). URL: https://bjs.ojp.gov/library/publications/prisoners-2019.

[3] Stephen Roberts, Jennifer Peirce, Sandhya Kajeepeta, and Madeline Bailey. "A Technical Guide to Jail Data Analysis". In: *Vera Institute of Justice* (2021). URL: https://www.vera.org/downloads/publications/a-technical-guide-to-jail-data-analysis-report.pdf.

[4] Brennan Klein. *Dataset on Incarcerated Populations in the United States*. Version 1.0.1. 2023. DOI: 10.5281/zenodo.7675566.

[5] Terry-Ann Craigie and Mariyana Zapryanova. "Mandatory minimum reforms, sentencing, and racial-ethnic disparities". In: *The B.E. Journal of Economic Analysis & Policy* 21.4 (2021), pp. 1289–1318. DOI: doi:10.1515/bejeap-2020-0215.

[6] David S. Lee and Justin McCrary. "The deterrence effect of prison: Dynamic theory and evidence". In: *Regression Discontinuity Designs*. Ed. by Matias D. Cattaneo and Juan Carlos Escanciano. Vol. 38. Advances in Econometrics. Emerald Publishing Ltd, 2017, pp. 73–146. URL: https://ideas.repec.org/h/eme/aecozz/s0731-905320170000038005.html.

[7] Stéphane Mechoulan and Nicolas Sahuguet. "Assessing racial disparities in parole release". In: *The Journal of Legal Studies* 44.1 (2015), pp. 39–74. DOI: 10.1086/680988.

[8] Angela E. Oh and Karen Umemoto. "Asian Americans and Pacific Islanders: From incarceration to re-entry". In: *Amerasia Journal* 31.3 (2005), pp. 43–60. DOI: 10.17953/amer.31.3.g01428017553275j.

[9] Veronica Horowitz and Christopher Uggen. "Consistency and compensation in mercy: Commutation in the era of mass incarceration". In: *Social Forces* 97.3 (2018), pp. 1205–1230. DOI: 10.1093/sf/soy065.

[10] Mary E. Campbell and Lisa Troyer. "The implications of racial misclassification by observers". In: *American Sociological Review* 72.5 (2007), pp. 750–765. DOI: 10.1177/000312240707200505.

[11] Ann Morning and Daniel Sabbagh. “From sword to plowshare: Using race for discrimination and antidiscrimination in the United States”. In: *International Social Science Journal* 57.183 (2005), pp. 57–73. DOI: https://doi.org/10.1111/j.0020-8701.2005.00531.x.

[12] Aliya Saperstein. “Capturing complexity in the United States: which aspects of race matter and when?” In: *Ethnic and Racial Studies* 35.8 (2012), pp. 1484–1502. DOI: 10.1080/01419870.2011.607504.

[13] Henry E. Brady and Cynthia S. Kaplan. “Conceptualizing and measuring ethnic identity”. In: *Measuring Identity: A Guide for Social Scientists*. Ed. by Rawi Abdelal, Yoshiko M. Herrera, Alastair Iain Johnston, and Rose McDermott. Cambridge University Press, 2009, pp. 33–71. DOI: 10.1017/CBO9780511810909.003.

[14] Jonathan Burton, Alita Nandi, and Lucinda Platt. “Measuring ethnicity: Challenges and opportunities for survey research”. In: *Ethnic and Racial Studies* 33.8 (2010), pp. 1332–1349. DOI: 10.1080/01419870903527801.

[15] Kristyn M. Bigback, Megan Hoopes, Jenine Dankovchik, Elizabeth Knaster, Victoria Warren-Mears, Sujata Joshi, and Thomas Weiser. “Using record linkage to improve race data quality for American Indians and Alaska Natives in two Pacific Northwest state hospital discharge databases”. In: *Health Services Research* 50.1 (2015), pp. 1390–1402. DOI: 10.1111/1475-6773.12331.

[16] Ioan Voicu. “Using first name information to improve race and ethnicity classification”. In: *Statistics and Public Policy* 5.1 (2018), pp. 1–13. DOI: 10.1080/2330443X.2018.1427012.

[17] Oregon Criminal Justice Commission. *Technical Documentation: Probabilistic race correction for Hispanics*. 2018. URL: https://www.oregon.gov/cjc/CJC%20Document%20Library/RaceCorrectionTechDocFinal-8-6-18.pdf.

[18] E. Ann Carson. “Prisoners in 2020 – Statistical Tables”. In: *Bureau of Justice Statistics* 302776 (2021). URL: https://bjs.ojp.gov/library/publications/prisoners-2020-statistical-tables.

[19] Florida Office of the State Courts Administrator. *Trial Court Statistics Search*. URL: http://trialstats.flcourts.org/.

[20] Ryan T. Cannon. “Sick deal: Injustice and plea bargaining during COVID-19”. In: *Journal of Criminal Law and Criminology* 110 (2020). DOI: 10.2139/ssrn.3632508.

[21] Thea Johnson. “Crisis and coercive pleas”. In: *Journal of Criminal Law and Criminology* 110 (2020). DOI: 10.2139/ssrn.3632052.

[22] Miko M. Wilford, David M. Zimmerman, Shi Yan, and Kelly T. Sutherland. “Innocence in the shadow of COVID-19: Plea decision making during a pandemic.” In: *Journal of Experimental Psychology: Applied* (2021). DOI: 10.1037/xap0000367.

[23] Ram Subramanian, Léon Digard, Melvin Washington II, and Stephanie Sorage. "In the shadows: A review of the research on plea bargaining". In: *Vera Institute of Justice* (2020). URL: https://www.vera.org/downloads/publications/in-the-shadows-pleabargaining.pdf.

[24] Arkansas Board of Corrections. *Modified EPA Update*. 2020. URL: https://ssl-adc.ark.org/modified-epa-update.

[25] John Pfaff. *Locked in: The true causes of mass incarceration—and how to achieve real reform*. Basic Books, 2017. ISBN: 0465096913.

[26] Perry Zurn, Danielle S. Bassett, and Nicole C. Rust. "The citation diversity statement: A practice of transparency, a way of life". In: *Trends in Cognitive Sciences* 24.9 (2020), pp. 669–672. DOI: 10.1016/j.tics.2020.06.009.

[27] Jordan D. Dworkin, Kristin A. Linn, Erin G. Teich, Perry Zurn, Russell T. Shinohara, and Danielle S. Bassett. "The extent and drivers of gender imbalance in neuroscience reference lists". In: *Nature Neuroscience* 23.8 (2020), pp. 918–926. DOI: 10.1038/s41593-020-0658-y.

[28] Paula Chakravartty, Rachel Kuo, Victoria Grubbs, and Charlton McIlwain. "#CommunicationSoWhite". In: *Journal of Communication* 68.2 (2018), pp. 254–266. DOI: 10.1093/joc/jqy003.

[29] Daniel Maliniak, Ryan Powers, and Barbara F. Walter. *The gender citation gap in international relations*. Vol. 67. 4. 2013, pp. 889–922. DOI: 10.1017/S0020818313000209.

[30] Michelle L. Dion, Jane Lawrence Sumner, and Sara Mc Laughlin Mitchell. "Gendered citation patterns across political science and social science methodology fields". In: *Political Analysis* 26.3 (2018), pp. 312–327. DOI: 10.1017/pan.2018.12.

[31] Neven Caplar, Sandro Tacchella, and Simon Birrer. "Quantitative evaluation of gender bias in astronomical publications from citation counts". In: *Nature Astronomy* 1 (2017). DOI: 10.1038/s41550-017-0141.

[32] Pierre Azoulay and Freda Lynn. "Self-citation, cumulative advantage, and gender inequality in science". In: *Sociological Science* 7 (2020). DOI: 10.15195/v7.a7.

[33] Gita Ghiasi, Philippe Mongeon, Cassidy R. Sugimoto, and Vincent Larivière. "Gender homophily in citations". In: *23rd International Conference on Science and Technology Indicators* (2018), pp. 1519–1525. URL: https://hdl.handle.net/1887/65291.

State	Primary source of data	Frequency	Data start	Data end
Alabama	Inmate Search website	weekly	Sep. 2000	Jan. 2022
Alaska	Research Records website	monthly	Jan. 2015	Jan. 2022
Arizona	Corrections at a Glance website	monthly	Jan. 2017	Jan. 2022
Arkansas	Director's Board Reports	monthly	Jan. 2000	Jan. 2022
California	Offender Population Reports and public records request	monthly	Jan. 2014	Jan. 2022
Colorado	Inmate Population Profile	monthly	Jun. 1992	Jan. 2022
Connecticut	Monthly Statistics	monthly	Jan. 2010	Jan. 2022
Delaware	Inmate Population Data	monthly	Jan. 2017	Jan. 2022
Washington D.C.	Inmate Demographics and Statistics	quarterly	Oct. 2015	Jan. 2022
Federal BOP	Inmate Race Statistics and public records request	weekly	Jan. 2011	Jan. 2022
Florida	Statistics and Publications and public records request	monthly	Jan. 2005	Jan. 2022
Georgia	Monthly Profile of All Inmates	monthly	Jan. 2006	Jan. 2022
Hawaii	Annual Reports and public records request	monthly	Mar. 2018	Jan. 2022
Idaho	Incarcerated Population Report and public records request	monthly	Jan. 2013	Jan. 2022
Illinois	Prison Population Data Sets	quarterly	Jun. 2005	Jan. 2022
Indiana	Fact Cards	quarterly	Jan. 2002	Jan. 2022
Iowa	Quarterly Quick Facts and public records request	monthly	Feb. 2005	Jan. 2022
Kansas	Population Reports	monthly	Jun. 1990	Jan. 2022
Kentucky	Monthly Reports	monthly	Dec. 2011	Jan. 2022
Louisiana	Annual Statistics and public records request	2x-yearly	Jan. 1989	Jan. 2022
Maine	Reports & Statistical Data	monthly	Jan. 2015	Jan. 2022
Maryland	Inmate Characteristics Reports and public records request	quarterly	Jul. 1990	Jan. 2022
Massachusetts	Institutional Fact Cards and Research Reports	monthly	Jan. 2012	Jan. 2022
Michigan	Statistical Reports	yearly	Jan. 1961	Jan. 2022
Minnesota	Historical Population Summary Reports	2x-yearly	Jan. 1998	Jan. 2022
Mississippi	Monthly Fact Sheets	monthly	Apr. 2001	Jan. 2022
Missouri	Publications and Information & Missouri Sunshine Law	monthly	Jun. 2004	Jan. 2022
Montana	Data & Statistics and public records request	monthly	Jan. 2005	Jan. 2022
Nebraska	Statistical Reports	quarterly	Dec. 2007	Jan. 2022
Nevada	Weekly Fact Sheets	weekly	Jan. 2015	Jan. 2022
New Hampshire	NHDOC Reports	monthly	Jul. 1999	Jan. 2022
New Jersey	Offender Stats	yearly	Jan. 2011	Jan. 2022
New Mexico	Notice & Reports and public records request	monthly	Jan. 2010	Jan. 2022
New York	Inmates Under Custody and public records request	monthly	Jan. 2000	Jan. 2022
North Carolina	DPS Research & Planning	quarterly	Jan. 1996	Jan. 2022
North Dakota	Data Narratives: Prison	monthly	Nov. 2001	Jan. 2022
Ohio	Monthly Fact Sheets	monthly	Aug. 2016	Jan. 2022
Oklahoma	Offender Information and public records request	weekly	Jul. 2010	Jan. 2022
Oregon	Inmate Population Profile and public records request	monthly	Jan. 1998	Jan. 2022
Pennsylvania	Monthly Population Reports and public records request	monthly	Feb. 2017	Jan. 2022
Rhode Island	Offender Statistics & Reports and public records request	monthly	Jan. 2012	Jan. 2022
South Carolina	Archived SCDC FAQS	monthly	Jul. 2011	Jan. 2022
South Dakota	Adult Inmates by Race/Ethnicity	monthly	Dec. 2016	Jan. 2022
Tennessee	Felon Population Reports	monthly	Jul. 2004	Jan. 2022
Texas	Inmate Population High Value Dataset and public records request	monthly	Sep. 2005	Jan. 2022
Utah	UDC Statistics and public records request	monthly	Sep. 2012	Jan. 2022
Vermont	Population Report	monthly	Jan. 2020	Jan. 2022
Virginia	Offender Population Reports	monthly	Jul. 2010	Jan. 2022
Washington	Statistical Reports	quarterly	Nov. 2009	Jan. 2022
West Virginia	Adult prison statistics and public records request	2x-yearly	Jul. 2002	Jan. 2022
Wisconsin	Data & Reports	monthly	Jul. 1990	Jan. 2022
Wyoming	Population Demographics Report and public records request	quarterly	Jan. 2006	Jan. 2022

Table A.1: Overview of Klein et al. dataset. This dataset includes prison population time series for all 50 states and the District of Columbia. Here, we have included hyperlinks to each state's website where we started to collect the data. For some states, we submitted public records requests in order to obtain data about inmate race statistics.

State	Date	Average pop. (Dec. ‘19 - Feb. ‘20)	Lowest pop. since Mar. ‘20	% of pre-pandemic pop.
New Jersey	2022-03-01	18,657	11,711	62.77%
New York	2022-02-01	45,201	30,451	67.37%
Washington	2022-03-01	19,134	13,221	69.10%
West Virginia	2021-01-01	5,798	4,007	69.11%
District of Columbia	2020-06-01	1,820	1,269	69.73%
Illinois	2022-04-01	38,797	27,183	70.06%
Maine	2022-01-01	2,235	1,579	70.65%
Connecticut	2021-05-01	12,633	8,948	70.83%
Massachusetts	2022-07-01	8,442	6,202	73.47%
Delaware	2021-06-01	5,887	4,381	74.42%
Vermont	2021-05-01	1,635	1,220	74.62%
North Dakota	2020-09-01	1,722	1,285	74.62%
California	2021-02-01	125,128	94,310	75.37%
New Hampshire	2022-08-01	2,640	1,997	75.64%
Rhode Island	2021-08-01	2,616	1,998	76.38%
Hawaii	2021-04-01	5,469	4,186	76.54%
Kentucky	2021-07-01	23,518	18,234	77.53%
Colorado	2021-07-01	19,877	15,434	77.65%
Minnesota	2021-07-01	9,354	7,369	78.78%
Arizona	2022-08-01	42,266	33,326	78.85%
Nevada	2022-01-01	13,149	10,394	79.05%
New Mexico	2022-08-01	7,115	5,626	79.07%
Maryland	2021-07-01	18,178	14,390	79.16%
Pennsylvania	2022-03-01	45,599	36,292	79.59%
Virginia	2022-02-01	29,378	23,599	80.33%
Tennessee	2022-01-01	29,940	24,532	81.94%
Texas	2021-05-01	142,483	116,926	82.06%
Utah	2020-12-01	6,669	5,483	82.22%
Oregon	2022-02-01	14,586	11,993	82.22%
Wisconsin	2021-07-01	23,492	19,361	82.42%
South Carolina	2022-05-01	18,236	15,056	82.56%
Kansas	2022-03-01	10,019	8,273	82.57%
North Carolina	2022-02-01	35,085	29,121	83.00%
Missouri	2021-03-01	27,439	22,778	83.01%
Florida	2021-12-01	94,611	78,593	83.07%
Oklahoma	2022-03-01	25,494	21,220	83.24%
Louisiana	2022-01-01	31,635	26,377	83.38%
South Dakota	2021-02-01	3,835	3,217	83.89%
Georgia	2021-04-01	55,047	46,201	83.93%
Michigan	2022-01-01	38,239	32,114	83.98%
Indiana	2022-02-01	26,796	22,620	84.42%
Federal	2021-02-01	176,431	151,328	85.77%
Idaho	2020-11-01	9,243	7,973	86.26%
Montana	2021-01-01	2,776	2,405	86.64%
Iowa	2020-09-01	8,483	7,375	86.94%
Wyoming	2021-01-01	2,454	2,134	86.96%
Ohio	2021-07-01	48,782	42,963	88.07%
Arkansas	2021-03-01	18,042	16,085	89.15%
Mississippi	2022-02-01	20,977	18,745	89.36%
Alabama	2021-03-01	27,989	25,106	89.70%
Alaska	2020-05-01	4,589	4,142	90.26%
Nebraska	2020-10-01	5,622	5,294	94.17%

Table A.2: Extent of prison population reductions. For each state, we list the date (after March 2020) that the prison population reached its lowest reported value, as well as the magnitude of each state's reduction (measured as the percent of pre-pandemic prison population). States are listed in ascending order by largest population reductions.

State	Source	BJS: State Prison Pop. (as of Dec. 31, '19)	Klein et al.: State Prison Pop. (as of Dec. 31, '19)	Difference
Texas	Demographic Highlights, On Hand [link]	158,429	142,169	16,260
Virginia	Monthly Population Summary [link]	36,091	29,233	6,858
Montana	Public records request; [link]	4,723	2,806	1,917
Florida	Public records request; [link]	96,009	94,197	1,812
Ohio	Monthly Fact Sheet [link]	50,338	48,697	1,641

Table A.3: Five states with the largest differences to BJS data. Because there were slight discrepancies between the data collected in the current study and the yearly data released by the BJS, we include this table, which links to the data sources used. These data were collected from states' Departments of Corrections websites (or obtained through Freedom of Information Act requests). For both of these states, the BJS includes a disclaimer. Montana: "Data for 2019 are not comparable to data for previous years." Ohio: "Includes a small number of incarcerated individuals sentenced to one year or less." For Texas and Virginia, we include the link to directly access data reported by the state for the time period in question, and in each case, the BJS data does not correspond to data reported by the state. The discrepancies in Florida's numbers are relatively small compared to the overall number of people in prison. As stated above, we are confident in the data collection here, which involved making successful public records requests to the Department of Corrections.

State	Method	Source	Relevant language
AL	×	×	×
AK	self-report	emailed	Race is self-identified by the inmate.
AZ	self-report	emailed	The data is self-reported/self-identifying.
AR	self-report	emailed	During the intake process, individuals self-report their race to staff.
CA	self-report	document	Ethnicity is self-reported by offenders who choose from a list of 28 ethnicity types.
CO	self-report	document	Data from the DOC were used... because it contained self-reported ethnicity for offenders.
CT	self-report	phone	Returned phone call to confirm the policy is self-report.
DE	self-report‡	document	e.g. "Delaware's Level IV population identified as..."
DC	×	×	×
FBOP	self-report	document	Race/ethnic categories are presented as separate... individuals can select multiple racial categories.
FL	self-report	phone	Returned phone call to confirm the policy is self-report.
GA	self-report	emailed	The data is self-reported at admission.
HA	self-report	emailed	Ethnicity is self-reported; PSD does not verify the accuracy of the information given.
ID	self-report	emailed	It is self-report within the pre-sentence investigation process.
IL	self-report	emailed	Self-report. IDOC does not verify the information.
IN	self-report	emailed	The offenders self-identify at the time of entry into our pre-determined categories (not free form).
IA	self-report	emailed	We collect from inmate self-identifying and court documents.
KS	self-report	emailed	Demographic info, including race, is collected as part of the intake process. Individuals self-identify.
KY	self-report	phone	Returned phone call to confirm the policy is self-report.
LA	×	×	×
ME	self-report	emailed	This information is self-reported by the resident during intake.
MD	self-report*	emailed	Both self-report and staff-assigned.
MA	self-report*	phone	It is mostly self-reported, but if the county sends a face sheet the Booking Officer will use that.
MI	self-report	emailed	Each inmate self-identifies to staff for the sake of collecting data that is used in statistical reports.
MN	self-report*	document	Race information may be self-identified or classified by an observer.
MS	self-report	emailed	Race is self-reported during the intake process.
MO	self-report	emailed	The offender is self-identifying.
MT	self-report	emailed	Self-reported, unless the individual refuses... [then] race would be estimated by staff during intake.
NE	self-report	emailed	The race data that we use for our statistical reports is self-reported by the inmate at intake.
NV	self-report‡	document	Demographic information is collected during intake... data are gathered from inmates progressively.
NH	self-report	emailed	The residents do self report upon intake. Thank you for your inquiry and have a great day.
NJ	self-report	emailed	Race and ethnicity are verified through self-report at intake.
NM	self-report	emailed	The inmates self-identify.
NY	self-report	document	The information about racial and ethnic origin is self-reported.
NC	self-report	emailed	Race is self-identified via a questionnaire that is administered during the entrance interview.
ND	self-report	emailed	Race information is self-reported.
OH	self-report‡	document	Information collected from incarcerated individuals: Race/ethnic origin, nationality, age...
OK	self-report	emailed	Yes, it's self-report by the inmate.
OR	self-report‡	document	HB 3064, passed August 2019, effective January 2020
PA	self-report	emailed	The PA DOC racial data is self-reported by the inmate at intake.
RI	self-report	document	...we cannot determine whether inmates identifying themselves as Hispanic are white or black.
SC	self-report	document	Based on inmate self-reported information at intake.
SD	self-report	document	...collect specified demographic information (religious preference, age, race, height, weight, etc.).
TN	self-report	emailed	The information is self-reported.
TX	visual	emailed	...during intake, the TDCJ will visually determine the race of the individual.
UT	self-report	emailed	Race is inmate self-identifying.
VT	self-report	document	Some categories are combined due to the low number of individuals who identify with these races...
VA	self-report	emailed	Virginia collects race information from several different sources, as well as being self-reported.
WA	self-report	document	Race and ethnicity are self-reported and grouped to include the following...
WV	self-report	emailed	No problem, in WV race is based on inmates self identification at intake.
WI	self-report	emailed	When an individual enters prison they are asked which race designation best describes them.
WY	self-report	phone	Returned phone call to confirm the policy is self-report.

∗ = Nonspecific policy; ‡ = Documentation is suggestive of self-report; × = could not confirm data.

Table A.4: Method of reporting race of incarcerated persons, by state. As of May 15, 2022, we have yet to receive confirmation from Alabama, District of Columbia, and Louisiana about their policies, but for nearly every state in this table, we see an explicit policy of recording racial categories via self-report.

Date	Total Defendants Filed	Total Disposed	Total Disposed Pre-trial	Pre-trial: Total Dismissed	Pre-trial: Total Guilty Plea	Pre-trial: Other
2017-07-01	14,883	12,442	12,199	1,094	9,604	1,501
2017-08-01	16,621	16,362	15,885	1,321	12,838	1,726
2017-09-01	12,109	10,461	10,148	913	8,043	1,192
2017-10-01	15,297	15,674	15,180	1,350	12,276	1,554
2017-11-01	13,848	13,799	13,403	1,131	10,748	1,524
2017-12-01	13,217	11,267	10,961	1,053	8,623	1,285
2018-01-01	15,752	14,322	13,845	1,408	10,908	1,529
2018-02-01	14,156	13,628	13,187	1,157	10,499	1,531
2018-03-01	15,309	13,956	13,579	1,145	10,837	1,597
2018-04-01	15,167	13,757	13,368	1,097	10,760	1,511
2018-05-01	15,590	14,643	14,170	1,228	11,332	1,610
2018-06-01	15,470	14,509	14,097	1,199	11,119	1,779
2018-07-01	15,265	14,080	13,874	1,324	11,150	1,400
2018-08-01	16,667	15,399	15,146	1,405	12,209	1,532
2018-09-01	13,475	13,233	13,046	1,204	10,488	1,354
2018-10-01	15,749	15,530	15,299	1,397	12,510	1,392
2018-11-01	13,904	13,634	13,433	1,210	10,744	1,479
2018-12-01	13,124	11,798	11,602	1,118	9,128	1,356
2019-01-01	15,431	14,839	14,665	1,430	11,444	1,791
2019-02-01	13,997	13,376	13,175	1,184	10,357	1,634
2019-03-01	14,423	14,440	14,237	1,364	10,990	1,883
2019-04-01	14,823	15,069	14,851	1,461	11,441	1,949
2019-05-01	15,973	15,054	14,627	1,615	11,052	1,960
2019-06-01	14,810	13,659	13,281	1,327	9,781	2,173
2019-07-01	15,004	14,689	14,311	1,523	11,340	1,448
2019-08-01	15,280	14,437	14,057	1,603	10,899	1,555
2019-09-01	12,629	12,855	12,477	1,435	9,827	1,215
2019-10-01	15,350	15,813	15,338	1,753	12,013	1,572
2019-11-01	11,901	12,720	12,379	1,354	9,691	1,334
2019-12-01	12,535	11,649	11,350	1,235	8,761	1,354
2020-01-01	13,715	14,590	14,212	1,483	10,914	1,815
2020-02-01	13,171	13,445	13,167	1,417	10,277	1,473
2020-03-01	14,335	9,935	9,789	1,121	7,412	1,256
2020-04-01	10,844	4,563	4,528	578	3,344	606
2020-05-01	10,783	4,542	4,513	582	3,229	702
2020-06-01	12,653	9,012	8,950	1,605	6,293	1,052
2020-07-01	12,018	8,054	8,036	1,123	5,928	985
2020-08-01	11,568	8,477	8,458	1,194	6,199	1,065
2020-09-01	12,279	9,467	9,441	1,190	7,148	1,103
2020-10-01	13,209	11,801	11,736	1,526	9,049	1,161
2020-11-01	11,280	9,817	9,747	1,189	7,582	976
2020-12-01	12,145	10,243	10,189	1,235	7,886	1,068
2021-01-01	11,862	9,648	9,624	1,262	7,242	1,120
2021-02-01	12,003	11,485	11,444	1,471	8,755	1,218
2021-03-01	13,689	14,149	13,993	1,720	10,748	1,525
2021-04-01	12,805	12,400	12,283	1,390	9,487	1,406
2021-05-01	12,351	12,518	12,393	1,437	9,492	1,464
2021-06-01	13,608	14,160	14,021	1,589	10,783	1,649

Table A.5: Statewide statistics for Florida Circuit Criminal Defendants. Data collected from the Trial Court Statistics Search (http://trialstats.flcourts.org/). Note the substantial drop-off in April and May of 2020 (highlighted above), corresponding to the period when Florida courts were closed or operating at highly reduced capacities.

State Prisons	Incarcerated Total			Incarcerated White			Incarcerated Black			Expected additional reduction in incarcerated Black population under White decarceration rates
	2019	2020	% diff	2019	2020	% diff	2019	2020	% diff	
Alabama	28,304	25,328	10.51%	13,454	11,607	13.73%	14,631	13,519	7.60%	–897
Alaska	4,475	4,578	–2.30%	1,863	1,850	0.70%	454	464	–2.20%	–14
Arizona	42,441	37,731	11.10%	16,523	14,613	11.56%	6,233	5,614	9.93%	–102
Arkansas	17,759	16,094	9.38%	9,767	8,691	11.02%	7,282	6,721	7.70%	–242
California	122,687	97,328	20.67%	25,510	18,819	26.23%	35,056	28,578	18.48%	–2,717
Colorado	19,785	16,168	18.28%	9,026	7,363	18.42%	3,507	3,016	14.00%	–156
Connecticut	12,823	9,559	25.45%	3,803	2,619	31.13%	5,457	4,201	23.02%	–443
Delaware	5,692	4,710	17.25%	1,932	1,493	22.72%	3,436	2,934	14.61%	–279
Florida	96,009	81,027	15.60%	38,335	31,647	17.45%	45,121	38,665	14.31%	–1,416
Georgia	54,816	47,141	14%	19,795	16,650	15.89%	32,656	28,406	13.01%	–939
Hawaii	5,279	4,171	20.99%	1,198	949	20.78%	323	186	42.41%	69
Idaho	9,437	8,171	13.42%	7,076	5,966	15.69%	287	267	6.97%	–26
Illinois	38,259	29,729	22.30%	12,223	9,271	24.15%	20,851	15,866	23.91%	–51
Indiana	27,180	23,944	11.91%	16,878	14,748	12.62%	8,852	7,888	10.89%	–154
Iowa	9,282	8,307	10.50%	6,047	5,350	11.53%	2,355	2,129	9.60%	–46
Kansas	10,177	8,779	13.74%	5,854	5,030	14.08%	2,755	2,399	12.92%	–32
Kentucky	23,082	18,552	19.63%	17,542	13,936	20.56%	4,877	4,052	16.92%	–178
Louisiana	31,609	26,964	14.70%	10,436	8,710	16.54%	21,061	18,143	13.85%	–566
Maine	2,185	1,714	21.56%	1,779	1,389	21.92%	231	178	22.94%	2
Maryland	18,595	15,623	15.98%	4,310	3,531	18.07%	13,197	11,120	15.74%	–309
Massachusetts	8,205	6,762	17.59%	3,462	2,805	18.98%	2,306	1,934	16.13%	–66
Michigan	38,053	33,617	11.66%	17,224	14,194	17.59%	20,040	17,231	14.02%	–717
Minnesota	9,982	8,148	18.37%	4,675	3,744	19.91%	3,586	2,994	16.51%	–123
Mississippi	19,417	17,577	9.48%	7,133	6,345	11.05%	12,025	10,998	8.54%	–302
Missouri	26,044	23,062	11.45%	16,286	14,405	11.55%	9,027	8,003	11.34%	–19
Montana	4,723	3,927	16.85%	3,359	2,778	17.30%	131	103	21.37%	5
Nebraska	5,682	5,306	6.62%	2,943	2,733	7.14%	1,559	1,454	6.74%	–7
Nevada	12,840	11,422	11.04%	5,537	4,830	12.77%	3,990	3,555	10.90%	–75
New Hampshire	2,691	2,352	12.60%	3,268	1,978	39.47%	140	159	–13.57%	–75
New Jersey	18,613	12,830	31.07%	3,978	2,619	34.16%	11,372	7,772	31.66%	–286
New Mexico	6,723	5,500	18.19%	1,687	1,477	12.45%	463	398	14.04%	7
New York	43,500	34,128	21.54%	10,421	7,685	26.25%	21,109	17,066	19.15%	–1,500
North Carolina	34,079	29,461	13.55%	13,553	11,614	14.31%	17,545	15,148	13.66%	–114
North Dakota	1,794	1,401	21.91%	1,098	848	22.77%	186	167	10.22%	–24
Ohio	50,338	45,036	10.53%	25,070	22,470	10.37%	21,752	19,454	10.56%	42
Oklahoma	25,679	22,462	12.53%	13,184	11,165	15.31%	6,689	5,940	11.20%	–276
Oregon	14,961	12,753	14.76%	10,757	9,257	13.94%	1,436	1,179	17.90%	56
Pennsylvania	45,702	39,357	13.88%	20,086	17,131	14.71%	20,846	18,240	12.50%	–461
Rhode Island	2,740	2,227	18.72%	1,142	853	25.31%	782	661	15.47%	–77
South Carolina	18,608	16,157	13.17%	6,950	5,896	15.17%	10,993	9,680	11.94%	–355
South Dakota	3,801	3,250	14.50%	1,998	1,727	13.56%	289	266	7.96%	–17
Tennessee	26,349	22,685	13.91%	14,633	12,359	15.54%	11,012	9,656	12.31%	–356
Texas	158,429	135,906	14.22%	53,597	45,596	14.93%	51,584	44,760	13.23%	–877
Utah	6,665	5,446	18.29%	4,048	3,245	19.84%	471	400	15.07%	–23
Vermont	1,608	1,284	20.15%	1,386	1,108	20.06%	141	123	12.77%	–11
Virginia	36,091	31,838	11.78%	15,005	13,266	11.59%	19,808	17,414	12.09%	98
Washington	19,261	15,724	18.36%	11,262	8,843	21.48%	3,275	2,777	15.21%	–206
West Virginia	6,800	6,044	11.12%	5,819	5,144	11.60%	875	802	8.34%	–29
Wisconsin	23,956	20,298	15.27%	10,815	8,879	17.90%	10,003	8,542	14.61%	–330
Wyoming	2,479	2,087	15.81%	1,864	1,591	14.65%	129	89	31.01%	21
**Nationwide**	**1,255,689**	**1,063,665**	**15.29%**	**515,591**	**430,817**	**16.44%**	**492,186**	**421,311**	**14.40%**	**–14,593**

Table A.6: Quantifying racial disparities in incarcerated population declines. Using yearly data from the Bureau of Justice Statistics [2, 18], we compare the incarcerated Black and White populations in each state from end-of-year 2019 to end-of-year 2020. The last column shows the expected additional decreases in incarcerated Black people if the Black and White incarcerated populations declined at the same rates.

State	Races included in incarcerated population reports
Alabama	Black, White, Other
Alaska	Alaska Native, Asian/Pacific Island, Black, Caucasian, Hispanic/Latino, Other/Unknown
Arizona	Hispanic, Caucasian, African-American, Native American, Other
Arkansas	Asian, Black, Hispanic, Native American Indian, Native Hawaiian or Pacific Islander, White, Other, Unknown
California	Hispanic, Black, White, Other
Colorado	Caucasian, Hispanic, African American, Native American, Asian
Connecticut	White, Black, Hispanic, American Indian, Asian
Delaware	Black, White, Asian or Pacific Islander, American Indian/Alaskan, Unknown
District of Columbia	Black, White, Hispanic, Other
Florida	White, Hispanic, Black, Other
Georgia	White, Black, Other, Asian, Unknown, Hispanic, Native American
Hawaii	American Indian, African American, Caucasian, Chinese, Filipino, Guam/Pacific Islander, Native Hawaiian, Hispanic, Japanese, Korean, Samoan, Other, Unknown
Idaho	Asian, Black or African American, Hispanic or Latino, Indian, Other, Unknown, White
Illinois	American Indian or Alaskan Native, Asian, African American or Black, Hispanic or Latino, Native Hawaiian or other Pacific Islander, White, Other, Two or More Races
Indiana	White, Black, Hispanic, Undetermined, American Indian/Alaskan Native, Asian/Pacific Islander
Iowa	White, Black, Hispanic, American Indian or Alaskan Native, Asian or Pacific Islander, Unknown
Kansas	White, Black, American Indian, Asian; reports Hispanic and Non Hispanic separately as ethnicity
Kentucky	White, Black, Hispanic, Bi-Racial, Asian, Native American, Other
Louisiana	White, Black, Other
Maine	Asian, Black or African American, Native American, Native Hawaiian, Two or More Races, Unknown, White
Maryland	Black, White, Latinx, Indian, Asian, Unknown
Massachusetts	Caucasian, African American, Hispanic, Asian, Other, Native American, Native Hawaiian/Pacific
Michigan	White, Non-White
Minnesota	White, Black, American Indian, Asian, Unknown/Other; reports Hispanic separately as ethnicity
Mississippi	Black, White, Hispanic, Native American, Asian, Data Unavailable
Missouri	Asian, Black, Hispanic, Native American, Unknown, White
Montana	American Indian/Alaska Native, Black, White, Asian/Pacific Islander
Nebraska	White, Black, Hispanic/Latino, American/Alaskan Native, Asian, Hawaiian/Pacific Islander, Other, Data Unavailable
Nevada	African American, American Indian, Asian, Caucasian, Hispanic, Other
New Hampshire	White, Black, Native American, Asian, Other/Unreported
New Jersey	Black, White, Hispanic, Asian, American Indian, Other or Not Coded
New Mexico	Asian, Black/African American, Hispanic Black, Hispanic Native Indian, Hispanic White, Native American/Alaskan, Native American, Pacific Islander, Unknown, White
New York	White, African American, Hispanic, Native American, Asian, Other, Unknown
North Carolina	White, Black/African American, American Indian/Alaskan Native, Asian/Asian American, Other, Unknown
North Dakota	Native American, Black, Hispanic, White, Other
Ohio	White, Black, Other Race
Oklahoma	African American, Caucasian, Hispanic, Native American, Other
Oregon	Asian, Black, Hispanic, American Indian, White, Unknown, Pacific Islander
Pennsylvania	Black, White, Hispanic, Other
Rhode Island	Black, White, Asian, Hispanic, Other
South Carolina	Black, White, Other
South Dakota	White, Native American, African American, Hispanic, Asian, Native Hawaiian/Pacific Islander
Tennessee	White, Black, Other
Texas	Black, White, Hispanic, Other and Unknown Race
Utah	Asian/Pacific Islander, Black, Hispanic, Native American/Alaskan, White, Unknown, White
Vermont	American Indian/Alaskan Native, Asian/Pacific Islander, Black or African American, White, Some Other Race/Unknown; reports Hispanic/Latinx/Spanish Identification separately as ethnicity
Virginia	Black, White, Other
Washington	White, Black, American Indian/Alaska Native, Asian/Pacific Islander, Other, Unknown
West Virginia	American Indian/Alaska Native, Asian, Black, Hispanic or Latino, Multi-Racial or Other, Native Hawaiian or Pacific Islander, White
Wisconsin	White, Black, American Indian/Alaskan Native, Asian or Pacific Islander, Unknown
Wyoming	Caucasian, African American, Hispanic, Native American, Other Race

Table A.7: States report race differently. In this table, the races listed (and their ordering) are copied exactly as reported by the state. All states include White or Caucasian as a race, most include Black or African American, many include Latino or Hispanic. Michigan reports White and Non-White as its race categories.
