# Supplementary material for: COVID-19 amplified racial disparities in the US criminal legal system
Source: Nature. 2023 Apr 19;617(7960):344–50. doi: 10.1038/s41586-023-05980-2 (PMC10172107; doi:10.1038/s41586-023-05980-2)
Supplement: Supplementary file 2 — Reporting Summary [file 41586_2023_5980_MOESM2_ESM.pdf]

nature portfolio

Corresponding author(s): Brennan Klein

Last updated by author(s): Feb 27, 2023

Reporting Summary

Nature Portfolio wishes to improve the reproducibility of the work that we publish. This form provides structure for consistency and transparency in reporting. For further information on Nature Portfolio policies, see our Editorial Policies and the Editorial Policy Checklist.

Statistics

For all statistical analyses, confirm that the following items are present in the figure legend, table legend, main text, or Methods section.

n/a	Confirmed	
☐	☒	The exact sample size (*n*) for each experimental group/condition, given as a discrete number and unit of measurement
☒	☐	A statement on whether measurements were taken from distinct samples or whether the same sample was measured repeatedly
☐	☒	The statistical test(s) used AND whether they are one- or two-sided<br>*Only common tests should be described solely by name; describe more complex techniques in the Methods section.*
☒	☐	A description of all covariates tested
☐	☒	A description of any assumptions or corrections, such as tests of normality and adjustment for multiple comparisons
☐	☒	A full description of the statistical parameters including central tendency (e.g. means) or other basic estimates (e.g. regression coefficient) AND variation (e.g. standard deviation) or associated estimates of uncertainty (e.g. confidence intervals)
☐	☒	For null hypothesis testing, the test statistic (e.g. *F*, *t*, *r*) with confidence intervals, effect sizes, degrees of freedom and *P* value noted<br>*Give P values as exact values whenever suitable.*
☒	☐	For Bayesian analysis, information on the choice of priors and Markov chain Monte Carlo settings
☒	☐	For hierarchical and complex designs, identification of the appropriate level for tests and full reporting of outcomes
☒	☐	Estimates of effect sizes (e.g. Cohen's *d*, Pearson's *r*), indicating how they were calculated

Our web collection on statistics for biologists contains articles on many of the points above.

Software and code

Policy information about availability of computer code

Data collection	We created a new dataset of the change in the prison population in all 50 states (and D.C.) over time. This involved a combination of web-scraping, public records requests, and manual data entry. The source of each state's data is documented in the manuscript and in the Github repository that contains the data. In this repository, we include time series of each prison system's population data. Additionally, we expanded on our analyses using datasets from states' departments of corrections, public records requests, and offices of the courts. The source, scope, and limitations of these datasets are documented in the text as they are introduced.
Data analysis	Data were analyzed using Python, and every analysis has a dedicated Jupyter notebook in the data repository explaining the various findings. This code is written in Python 3.7 and uses the following packages: Pandas 1.1.3, tabula 2.2.0, Numpy 1.19.2, beautifulsoup4 4.9.3, requests 2.24.0. Additionally, we assembled a series of tutorials about how to scrape pdf tables from public sources. Repository: https://github.com/jkbren/incarcerated-populations-data. This repository (Version 1.0.1.) is archived under the following Zenodo doi, 10.5281/zenodo.7675566. Note: the COMPAS risk assessment tool was referenced in the main text but is not used in this study.

For manuscripts utilizing custom algorithms or software that are central to the research but not yet described in published literature, software must be made available to editors and reviewers. We strongly encourage code deposition in a community repository (e.g. GitHub). See the Nature Portfolio guidelines for submitting code & software for further information.

Data

Policy information about availability of data

All manuscripts must include a data availability statement. This statement should provide the following information, where applicable:

- Accession codes, unique identifiers, or web links for publicly available datasets
- A description of any restrictions on data availability
- For clinical datasets or third party data, please ensure that the statement adheres to our policy

The incarceration data used in this work are public records in each state, and we have included the source urls in Table A.1. Together, data from all 50 states, the District of Columbia, and the Federal Bureau of Prisons create the "The Dataset on Incarcerated Populations", which we have made publicly available via an archived Zenodo repository, doi: 10.5281/zenodo.7675566 as well as through a Github repository (https://github.com/jkbren/incarcerated-populations-data). The source data used to construct the Dataset on Incarcerated Populations is available via direct download through the links provided in Table A.1, by public records request, or by request to the corresponding author(s).

Human research participants

Policy information about studies involving human research participants and Sex and Gender in Research.

Reporting on sex and gender	This study did not involve experimentation on human subjects -- instead, population-level summaries of incarcerated populations were used to generate the observational findings reported in this manuscript.
Population characteristics	While there were not human subject experiments in this work, we sought out data specifically about incarcerated persons' race in every state.
Recruitment	N/A
Ethics oversight	Because we use aggregated, already existing data in this manuscript, formal review was not sought.

Note that full information on the approval of the study protocol must also be provided in the manuscript.

Field-specific reporting

Please select the one below that is the best fit for your research. If you are not sure, read the appropriate sections before making your selection.

☐ Life sciences ☒ Behavioural & social sciences ☐ Ecological, evolutionary & environmental sciences

For a reference copy of the document with all sections, see nature.com/documents/nr-reporting-summary-flat.pdf

Behavioural & social sciences study design

All studies must disclose on these points even when the disclosure is negative.

Study description	This study is largely observational, identifying the signature of structural disparities in policy based on race using public records. We propose several hypotheses about potential mechanisms that could bring about the observed trends, and we use publicly available data to offer evidence for each mechanism.
Research sample	States' Departments of Correction release periodic statistical summaries about the total population of incarcerated people in the state prison system. We have data from 50 states, the District of Columbia, and the Federal Bureau of Prisons, spanning nearly 20 years. Table A.1 in the Supplementary Information details precisely the duration and reporting frequency of the data from various states' prison systems.
Sampling strategy	For each state, we collected the most temporally-resolved data that the state had. For most states in the dataset, we have monthly counts of the number of incarcerated people, by race and sex. For several, we have this data at the weekly level, and three states only report data at the yearly level. This procedure is detailed in Section A.1 of the Supplementary Information, as well as the "State prison populations over time" subsection of the Data & Methods section.
Data collection	Data were collected by the coauthors either manually (i.e., direct input from an online source or using web-scraping tools) or through public records requests (i.e., Freedom of Information requests).
Timing	The study period is primarily between March 2020 and January 2022, but for proper comparison with historical trends, we analyze time series data since 2013.
Data exclusions	N/A

Non-participation	N/A
Randomization	N/A

Reporting for specific materials, systems and methods

We require information from authors about some types of materials, experimental systems and methods used in many studies. Here, indicate whether each material, system or method listed is relevant to your study. If you are not sure if a list item applies to your research, read the appropriate section before selecting a response.

Materials & experimental systems

n/a	Involved in the study
☒	☐ Antibodies
☒	☐ Eukaryotic cell lines
☒	☐ Palaeontology and archaeology
☒	☐ Animals and other organisms
☒	☐ Clinical data
☒	☐ Dual use research of concern

Methods

n/a	Involved in the study
☒	☐ ChIP-seq
☒	☐ Flow cytometry
☒	☐ MRI-based neuroimaging
